# Metagenomic insights into rhizosphere microbial communities and functional gene profiles associated with the responses of tea yield and quality to nitrogen-zinc co-fertilization

**DOI:** 10.3389/fpls.2026.1852312

**Published:** 2026-07-10

**Authors:** Min Lu, Dandan Qi, Qiong Wang, Xin Sun, Yali Shi, Xiaojia Zhang, Ying Feng, Xiaoe Yang, Lubin Song, Chunwang Dong, Changbo Yuan

**Affiliations:** 1 State Key Laboratory of Nutrient Use and Management, Shandong Engineering Research Center of Tea Biology and Resource Utilization, Tea Research Institute, Shandong Academy of Agricultural Sciences, Jinan, China; 2 School of Energy, Environmental and Geomatics Engineering, Anqing Normal University, Anqing, China; 3 Key Laboratory of Environmental Remediation and Ecosystem Health, Ministry of Education (MOE), College of Environmental and Resources Sciences, Zhejiang University, Hangzhou, China

**Keywords:** functional genes, metagenomics, microbial co-occurrence networks, N-Zn co-fertilization, tea quality, tea yield

## Abstract

Optimal co-fertilization of nitrogen (N) and zinc (Zn) offers a promising approach for promoting the growth of tea plant (*Camellia sinensis* (L.) O. Kuntze), sustaining stable yield, and improving tea quality. However, the specific roles of rhizosphere microorganisms in mediating the tea yield and quality after N-Zn co-fertilization remain unclear. Here, a field experiment was carried out to assess the influence of N-Zn co-fertilization on the growth of tea plant, as well as the structure and functions of rhizosphere microbial communities in tea plantations. Results showed that N application contributed more to the increment of tea yield than Zn fertilization, whereas Zn supply significantly promoted the synthesis of free amino acids and reduced tea polyphenol contents as well as TP/AA at moderate N level. Zn addition decreased the level of soil NO_3_^−^‐N but increased NH_4_^+^-N concentrations at both moderate and high N levels. Soil metagenomic sequencing indicated that Zn supply significantly increased the relative abundances of microbial taxa involved in denitrification, such as *Arthrobacter*, *Bacillus*, *Terrabacter* and *Burkholderia*, as well as up-regulated the relative abundances of *narH*, *nasA*, *nasB*, *napB*, *nirB*, *nirD* and *norB* genes at high N level, which are related to some metabolic potential pathways like denitrification and nitrate reductase. Partial least squares path models showed that fertilization initially altered soil properties and enzyme activities, thereby affecting rhizosphere microbial communities and functional gene profiles, which sequentially contributed to the nutrient accumulation in tea plants and ultimately influenced tea yield. Random forest analysis further identified soil properties such as pH, OM, AP, NH_4_^+^-N, NO_3_^−^-N and AZn as the most influential factors affecting tea yield and quality. Overall, our results highlight the relationship between tea yield and quality with rhizosphere microbial communities and functional gene profiles under different N-Zn co-fertilizations. All these findings provide new perspective for nutrient use and management in tea plantations.

## 1 Introduction

As a globally significant cash crop, tea plant (*Camellia sinensis* (L.) O. Kuntze) owes its commercial importance to the unique sensory and bioactive properties that drive consumer demand (Ma et al., 2021). China’s tea sector has rapidly scaled to meet this demand, accounting for 62.1% of global cultivation area and 47.6% of production in 2020 (FAO, 2020). In this context, fertilization practices critically influence yield and quality outcomes. Yet, in the absence of precise nutrient recommendations, many tea plantations exhibit rampant over-application of synthetic fertilizers, a short-term yield strategy that ultimately suppresses tea quality, reduces long-term productivity, and induces environmental degradation such as soil structural decline and aquatic eutrophication (Ye et al., 2022). Thus, developing appropriate fertilization protocols represents a key step toward reconciling productivity goals with ecological sustainability in tea cultivation.

Nitrogen (N) fertilization is fundamental to tea production, as tea plants exhibit a significantly higher N demand compared to many other crops (Tang et al., 2022). Optimal N application effectively enhances both tea yield and quality (Ma et al., 2021). This is because N, a pivotal constituent of proteins, nucleic acids, and chlorophyll, actively stimulates the biosynthesis of C- and N-containing compounds. An adequate N supply promotes the accumulation of amino acids (notably theanine), supports balanced lipid metabolism, and elevates the production of key flavor metabolites, all of which are critical determinants of superior tea aroma and taste (Ruan et al., 2010). Conversely, excessive N can inhibit plant growth and impair quality; for instance, it may lead to over-accumulation of arginine, which is associated with undesirable bitter notes. Therefore, improving soil N availability and plant N utilization efficiency is paramount for optimizing tea productivity and quality. In soil, N availability is governed by a complex network of microbially driven transformation processes, including mineralization, nitrification, denitrification, dissimilatory nitrate reduction to ammonium and so on (Kuypers et al., 2018), which are mediated by specific functional microbes such as ammonia-oxidizing, nitrifying and denitrifying bacteria (Carey et al., 2016). Recently, emerging evidence suggests that zinc (Zn) can mediate N cycling and influence the activity of N assimilation enzymes in plants (Lv et al., 2022; Xu et al., 2024). This implies that soil Zn status may be a key factor limiting N use efficiency in tea plantations, presenting a potential key point for nutrient management.

Zn, an essential micronutrient, serves not only as a structural or catalytic component for enzymes involved in hydrolysis, redox reactions, and protein synthesis in tea plants (Zhang et al., 2017), but also as a key modulator of broader nutrient cycles. Its role as a cofactor for enzymes critical to N metabolism underpins an observed synergy between N and Zn in agricultural systems (Glass and Orphan, 2012). For instance, in rice, combined N and Zn application upregulates specific transporter genes, enhancing root-to-shoot translocation and partitioning of both nutrients, thereby increasing grain yield (Ji et al., 2022). A similar interaction in tea is suggested by the function of CsZIP4 in mediating both Zn transport and N utilization (Xu et al., 2024). Beyond these direct plant physiological interactions, Zn can indirectly influence N availability via the soil microbiome. Elevated Zn levels have been shown to directly inhibit key N-cycling microbes, such as nitrifiers and nitrogen-fixing bacteria, and their associated enzymes, thereby suppressing processes like nitrification (Ruyters et al., 2010). Although Zn-mediated effects on soil N turnover have been reported in other systems, whether Zn fertilization can positively modulate soil N transformation processes in the unique background of tea plantation remains unclear.

Rhizosphere microbiome, often regarded as a plant’s “second genome”, establishes functionally rich symbioses crucial for enhancing host nutrition and fitness (Trivedi et al., 2020). These interactions effectively extend the plant’s capacity for growth, nutrient acquisition, and quality development without altering its genetic code (Bahram et al., 2018). For example, inoculations with arbuscular mycorrhizal fungi and plant growth-promoting rhizobacteria can enhance root colonization, leading to improved N uptake in tea plants (Cao et al., 2024). Soil microbial community composition is highly sensitive to soil conditions altered by fertilization and other management practices (Yu et al., 2023; Wang et al., 2025). Consequently, fertilization-induced shifts in microbial diversity and structure can indirectly influence tea yield and quality (Ma et al., 2021; Tang et al., 2022, 2023; Yin et al., 2025). Beyond community structure, the abundance of specific microbial functional genes offers a direct window into how environmental changes affect ecosystem processes. For instance, intercropping with leguminous green manure in tea plantations was shown to downregulate nitrification genes, thereby reducing N loss and promoting theanine accumulation (Duan et al., 2023). While integrating microbial drivers into soil-plant research is increasingly emphasized, the precise mechanisms through which key functional genes regulate ecosystem functions and plant productivity under N-Zn co-fertilization in tea plantations remain unexplored.

Recent study by Wang (2025) has reported the effects of different N-Zn combined treatments on tea plant growth, key enzyme activities in N metabolism, and fresh tea leaf quality. Additionally, our previous work has evaluated the effects of different N and Zn fertilization combinations on N availability, bacterial communities and metabolomics in the rhizosphere soil of tea plants (Lu et al., 2025). However, limited information is available regarding the underlying mechanisms involved in soil N cycling and Zn resistance in the rhizosphere of the tea plants, and the aboveground characteristics in response to N-Zn co-fertilization patterns. To address these knowledge gaps, this study investigated variations in the relative abundances (RAs) of functional genes associated with soil N cycling and Zn resistance through metagenomic analysis, comparing the effects of different fertilization regimes on soil microbial communities under combined N and Zn application over a field experiment. Therefore, the aims of this study were to i) characterize the responses of tea yield, quality, and nutrient contents in tea leaves; (ii) elucidate the different changes in key microbial taxa and the specific functional genes involved in soil N cycling and Zn resistance and (iii) preliminarily explore the potential associations of soil properties, soil microbial communities with tea yield and quality.

## 2 Materials and methods

### 2.1 Field description and experimental design

The tested tea plantation was provided by Shandong Lingquan Tea Industry Co., Ltd from 2023-2024, which is located in Chengqian town, Zoucheng City, Shandong Province (35°09′-35°32′ N, 116°44′-117°28′ E). The experimental site has a temperate monsoon climate, with an average annual precipitation of 687 mm and an annual mean temperature of 14.9 °C. The initial physicochemical characteristics of the soil were as follows: pH 5.62, organic carbon (OM) content 4.36 g/kg, total nitrogen (TN) content 0.649 g/kg, available phosphorus (AP) content 41.8 mg/kg, and available potassium (AK) content 72 mg/kg. The tea cultivar Zhongcha108, which is a variety suitable for producing premium green tea, had been growing on the experimental site for 8 years prior to this study. Row spacing in the experimental plot was 0.5 m, and plant spacing was 0.4 m. Based on the previous researches (Zhao, 2016; Ma et al., 2021), the experimental treatments were set up as follows: (i) CK: control, 119 kg N/ha (moderate N level) and 3 mg Zn/kg soil (moderate Zn level); (ii) Zn treatment: 119 kg N/ha (moderate N level) and 12 mg Zn/kg soil (high Zn level); (iii) N treatment: 569 kg N/ha (high N level) and 3 mg Zn/kg soil (moderate Zn level); and (iv) N+Zn treatment: 569 kg N/ha (high N level) and 12 mg Zn/kg soil (high Zn level). N was applied as urea and was split into two different applications: 60% in October 2023 and 40% in March 2024. Zn was applied in the form of zinc sulfate as a basal fertilizer in October 2023. For all treatments, phosphorus (P) and potassium (K) were applied in October as superphosphate (39.3 kg P/ha) and potassium sulfate (99.6 kg K/ha). Fertilizers were applied manually at a depth of 10–20 cm to the inter-rows and covered with soil afterward. The four treatments were set up with four replicates based on a random block design, and each replicate was conducted in a 20 m^2^ (2 m × 10 m) experimental plot.

### 2.2 Tea sampling, nutrient element determination, and quality analysis

Tea shoots (one bud and two young expanding leaves) were harvested by hand in 20th April 2024 as spring tea. The tea yield was calculated using a 30 × 30 cm sampling frame, by estimating the number of one bud and two young expanding leaves units within the frame and the weight of 100 tea buds. Six sampling replicates were there for each experimental plot, each separated by 2.5 m. Concurrently, root samples were collected from the tea plants. Both the harvested shoots and roots were oven-dried and ground into a fine powder. Subsequently, the dried samples were subjected to digestion with 5 mL H_2_SO_4_ (98%) and several drops of H_2_O_2_ on a heater at up to 180 °C. The concentrations of total N in the digestion solution were determined using a flow injection analyzer (FIAstar 5000, Sweden), and the Zn concentrations in the digestion solution were determined using an inductively coupled plasma mass spectrometer (ICP-MS, 7500a, Agilent, USA). The translocation factors (*TF*) were calculated according to the following formulas: $TF=c_{leaf}/c_{root}$. To assess tea quality, the dried samples of tea shoots were extracted with boiling distilled water (ddH_2_O) for 5 minutes. The concentration of total free amino acid (AA) in the extracts was determined using glutamic acid as a reference standard by the ninhydrin colorimetric method at 570 nm (Ruan et al., 2007; Guo et al., 2025a) described in GB/T8314-2013. For the determination of total polyphenols (TP) content, the dried samples of tea shoots were extracted with 70 °C of 70% MeOH for 10 minutes. The TP content in the extracts was measured using gallic acid as the reference standard by the Folin-Ciocalteu colorimetric method at 765 nm (Guo et al., 2025b; Pita-Barbosa et al., 2025) described in GB/T8313-2018. The details of the analysis procedures were given in the **Supplementary Materials**.

### 2.3 Soil sample collection, physicochemical properties analysis and enzyme activity determination

Rhizosphere soils were sampled based on the methods of Edwards et al. (2015) with some minor modifications. Topsoil (0-20 cm deep) samples from four replicate plots per treatment were collected. Soil samples were immediately placed in sterile plastic containers and sent to the laboratory. The soil samples were sieved (2 mm mesh) and thoroughly homogenized into three portions. One portion was air-dried, ground and passed through a 1 mm sieve for soil characterization. The second portion was kept at 4 °C for 5 days for enzyme activities analysis and extraction of microbial biomass carbon (MBC) and nitrogen (MBN), soil ammonium nitrogen (NH_4_^+^-N) and nitrate nitrogen (NO_3_^−^-N). The rest was stored at −80 °C for 7 days for DNA extraction. Soil physicochemical properties were determined by standard procedures characterized by Bao (2008) and the details of the analysis procedures were given in the **Supplementary Materials**. Soil MBC and MBN contents were determined by chloroform fumigation followed by K_2_SO_4_ extraction with a TOC analyzer (multi-C/N 3100, Analytik Jena AG, Jena, Germany). Soil urease (S-UE) activity was determined with the sodium phenol-sodium hypochlorite colorimetric method (Zhang et al., 2011). Soil sucrase (S-SC) activity was measured with the 3,5-dinitrosalicylic acid colorimetric specific terms (Schinner and VonMersi, 1990). Measurement of soil acid phosphatase (S-ACP) activity was conducted by the ρ-nitrophenyl-phosphate method (Guan, 1986). Soil peroxidase (S-POD) activity was determined with the autoxidation of the pyrogallol oxidation method. Soil polyphenol oxidase (S-PPO) activity was measured based on the pyrogallol colorimetry method.

### 2.4 Soil metagenomic sequencing analysis

0.2g of soil was used to extract total genomic DNA with the he E.Z.N.A.^®^ soil DNA Kit (Omega Bio-tek, Norcross, GA, U.S.) according to manufacturer’s instructions. Concentration and purity of extracted DNA was determined with SynergyHTX and NanoDrop2000, respectively. DNA quality was checked on 1% agarose gel. DNA fragments, averaging 350 bp in length, were prepared using the Covaris M220 (Gene Company Limited, China). Paired-end sequencing libraries were constructed using the NEXTFLEX^®^ Rapid DNA-Seq Kit (Bioo Scientific, Austin, TX, USA), attaching sequencing primer hybridization sites. Sequencing was performed on the Illumina NovaSeq™ X Plus platforms (Illumina Inc., San Diego, CA, USA), following the manufacturers’ guidelines with the NovaSeq™ X Series 25B Reagent Kit. The raw sequencing reads were trimmed of adapters, and low-quality reads (length < 50 bp or with average quality value < 20) were removed by fastp (https://github.com/OpenGene/fastp, version 0.20.0) (Chen et al., 2018). Macrogenome assembly was performed using MEGAHIT (version 1.1.2), with contig sequences shorter than 300 bp filtered out (Wang et al., 2025). Open reading frames (ORFs) from each assembled contigs were predicted using Prodigal (version 2.6.3) and a length larger than 100 bp ORFs were retrieved (Xue et al., 2025). Redundancy was removed using CD-HIT (version 4.7) with 90% sequence identity and coverage. Gene abundance for a certain sample was estimated trough Reads Per Kilobase per Million mapped reads (RPKM) by SOAPaligner (http://soap.genomics.org.cn/, version soap2.21 release) with 95% identity. Quality control of the raw sequencing reads yielded 655,032,750 clean reads, with an average of 40,939,546.88 reads per sample, which were used for subsequent bioinformatics analyses. The best-hit taxonomy of non-redundant genes was achieved through aligning them against the NCBI NR database by DIAMOND (http://ab.inf.uni-tuebingen.de/software/diamond/) with an e-value cutoff of 1e^−5^ (Wang et al., 2025). KEGG annotation was performed using DIAMOND against the KEGG database (http://www.genome.jp/kegg/) with an e-value cutoff of 1e^−5^ (Yang et al., 2023). Genes related to Zn resistance were identified using HMMER with the BacMet database (http://bacmet.biomedicine.gu.se/) with an e-value cutoff of 1e^−5^ (Zhu et al., 2025). All generated data were deposited in the National Center for Biotechnology Information (NCBI) Sequence Read Archive under Bioproject PRJNA1395687 with BioSample accession numbers SAMN4369795 to SAMN4369810.

### 2.5 Statistical analysis

The normality and homogeneity of the dataset were assessed using the Shapiro-Wilk test together with Q-Q plots and Levene’s test, respectively. Statistical analyses were conducted using SPSS 20.0 and Origin 8.5 based on one-way ANOVA analysis at a significance level of *P* < 0.05. Duncan’s Multiple Range Test (DMRT) was applied for multiple comparison procedures at 5% and 1% significance levels, respectively. Differences in microbial community structures were analyzed using non-metric multidimensional scaling (NMDS) and permutational multivariate analysis of variance (PERMANOVA) based on the Bray-Curtis dissimilarities. The RAs of genes corresponding to orthology numbers were determined through comparison across all Kyoto Encyclopedia of Genes and Genomes orthology numbers. The RAs of Zn resistance genes screened from the BacMet database, were determined based on the total numbers of BacMet genes present in a sample. Variance inflation factor (VIF) analysis and redundancy analysis (RDA) were implemented using the “vegan” package to investigate the contribution of soil properties to microbial communities. Hierarchical partitioning analysis and significant testing were performed using the “rdacca.hp” package to evaluate the explanatory variance and significance of various explanatory variables for genes related to N cycling and Zn resistance, respectively (Lai et al., 2022). Partial least squares path modeling (PLS-PM) was performed using the “plspm” package. Random forest analysis was performed using the “randomForest” package and “rfPermute” packages (Liao et al., 2024). Normalized stochasticity ratio (NST) was performed using “nst” package to implement null model analysis (Ning et al., 2019). Microbial co-occurrence networks were constructed to reveal the complex microbiome interactions under different fertilization treatments. To minimize the occurrence of rare genera, only genera present in > 80% of the samples were preserved as persistent genera. Then, top 500 most abundant microbial genera of the persistent genera were selected to generate networks to avoid spurious correlations. Pearson correlation coefficients |r| > 0.6 and BH-adjusted *P* < 0.05 were selected for constructing the co-occurrence network. Gephi (http://gephi.github.io/) was used to generate topological features and for network visualization. Nodes were categorized as peripherals (Zi < 2.5 and Pi < 0.62), connectors (Zi < 2.5 and Pi > 0.62), module hubs (Zi > 2.5 and Pi < 0.62), and network hubs (Zi > 2.5 and Pi > 0.62). Random attack strategy was adopted to perform robustness testing of the co-occurrence networks through node removal simulation using “igraph” package.

## 3 Results

### 3.1 Tea yield and quality

**Figure 1:**
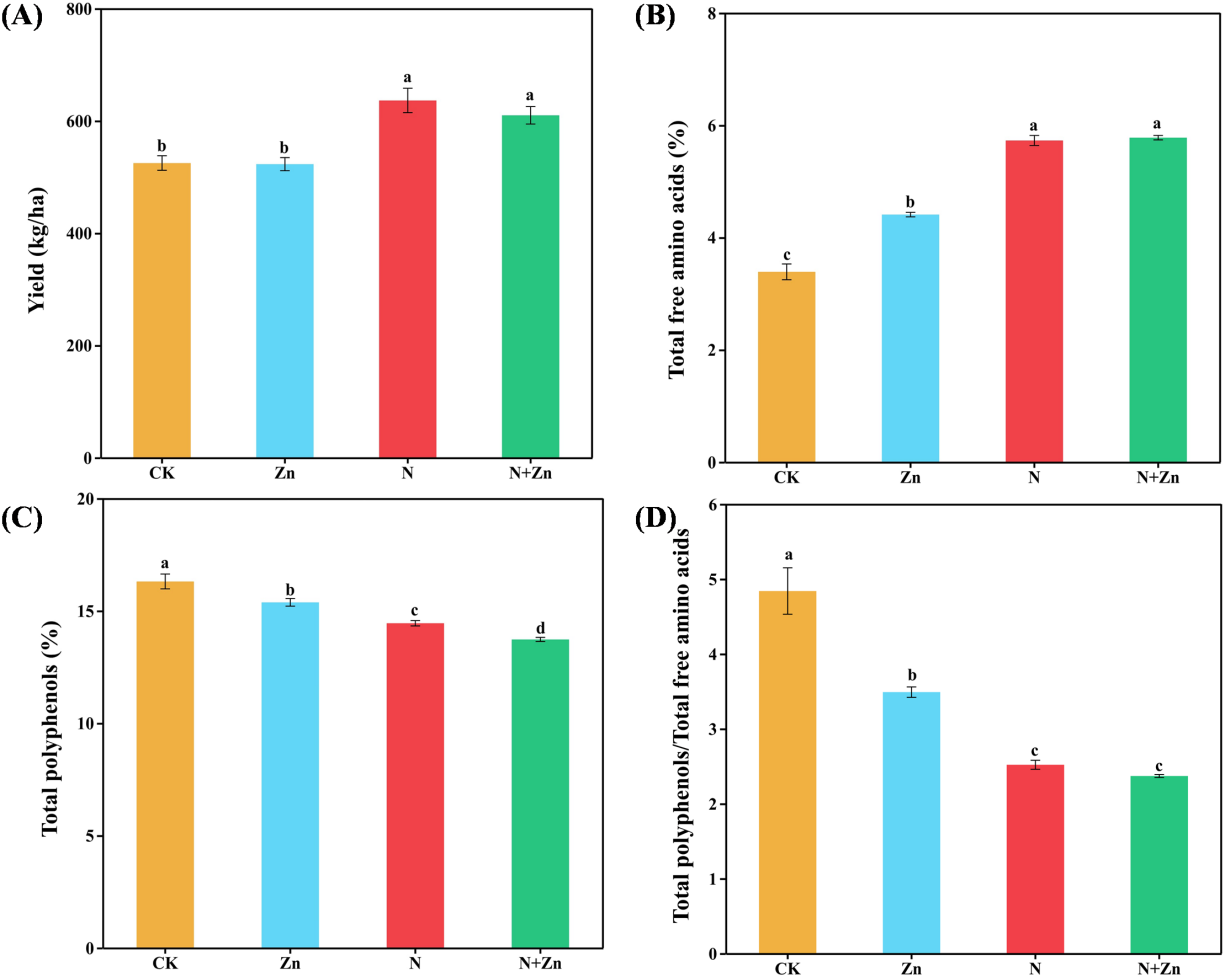
Tea yield **(A)**, total free amino acids contents **(B)**, total polyphenols contents **(C)** and tea polyphenols/total free amino acids **(D)** in response to different combinations of N and Zn fertilization. Data represent the means ± standard errors (n = 4). Different letters above the bars indicate significant differences (*P* < 0.05) among all fertilization treatments. CK, control; Zn, Zn treatment; N, N treatment; N+Zn, N+Zn treatment. The same is below.

Tea yields were significantly increased after both N and N+Zn treatments compared to CK treatment, while there was no significant change under Zn fertilization (**Figure 1A**). N fertilization significantly increased tea yield by 21.2%. However, N-Zn co-fertilization reduced this increment, resulting in a 16.2% yield increase compared to the CK treatment. It is noted that Zn supply did not show significant impact on tea yield at both moderate and high N levels. Fertilization significantly influenced tea quality, leading to an increase in AA content, a decrease in TP content, and a reduction in the ratio of TP to AA (TP/AA). After Zn, N and N+Zn treatments, the AA content significantly increased by 30.2%, 69.3% and 70.8%, respectively (**Figure 1B**), the TP content significantly decreased by 5.69%, 11.4% and 15.8%, respectively (**Figure 1C**), TP/AA significantly reduced by 27.9%, 47.9% and 32.0%, respectively (**Figure 1D**), as compared to CK treatment. And Zn addition obviously promoted AA content, reduced TP content and decreased TP/AA at moderate N level.

### 3.2 N and Zn concentrations in different tissues of the tea plants

**Figure 2:**
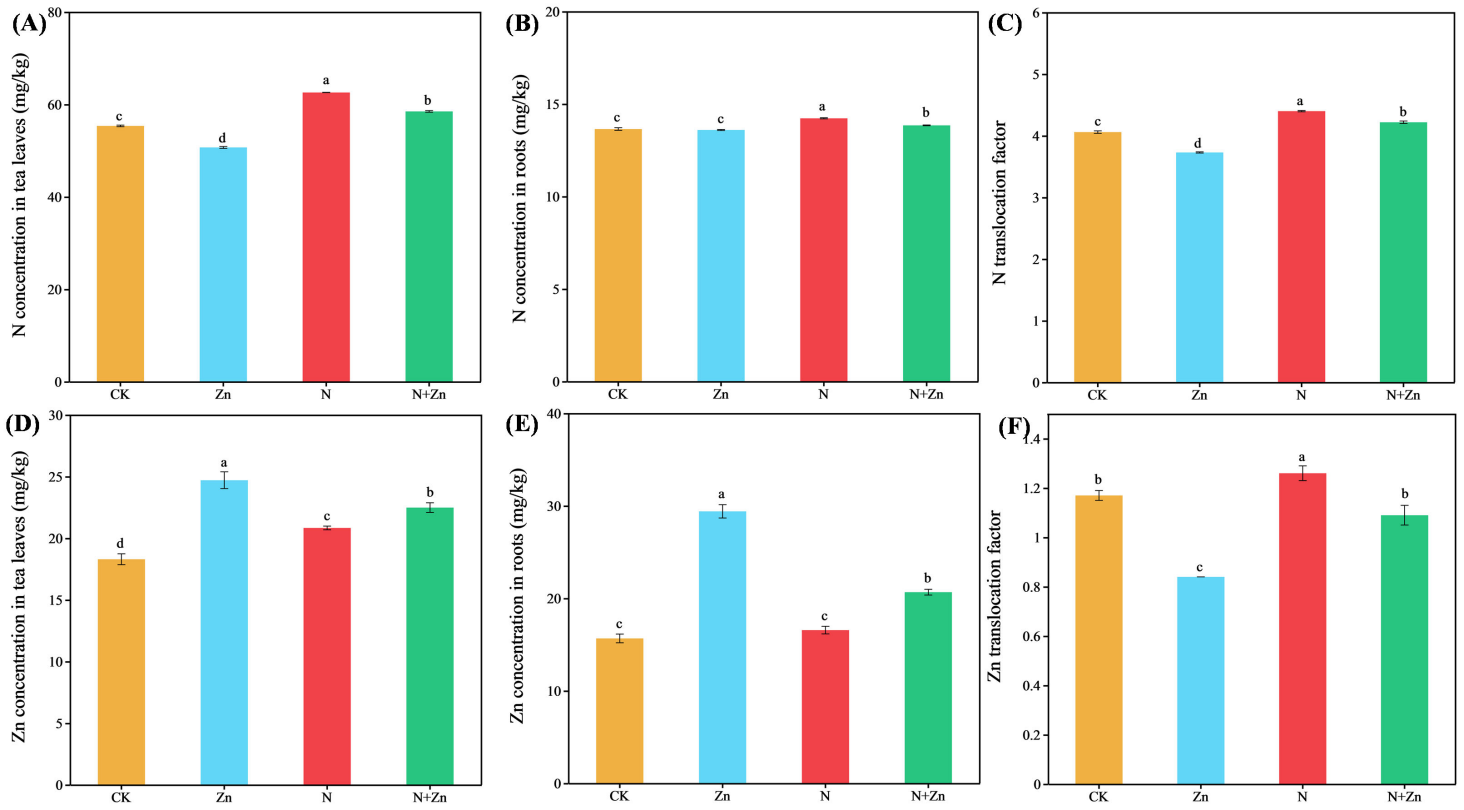
**N** concentration in tea leaves **(A)**, N concentration in roots **(B)**, N translocation factor from root to tea leaves **(C)**, Zn concentration in tea leaves **(D)**, Zn concentration in roots **(E)**, Zn translocation factor from root to tea leaves **(F)** in response to different combinations of N and Zn fertilization. Data represent the means ± standard errors (n = 4). Different letters above the bars indicate significant differences (*P* < 0.05) among all fertilization treatments.

Fertilization had significant impact on the N and Zn concentrations in roots and tea shoots (**Figure 2**). Apart from root N concentration at moderate N level, Zn supply significantly decreased root N concentration at high N level (2.67%) (**Figure 2B**) and the N concentrations in tea leaves at both moderate and high N levels (8.42% and 6.54%) (**Figure 2A**). However, Zn concentrations in roots and tea shoots were markedly promoted after Zn supply at both moderate and high N levels, which increased by 87.7% and 24.7%, 35.0% and 7.93%, respectively (**Figures 2D, E**). Notably, the increments at moderate N level were larger than those at high N level. In addition, contrast to Zn concentrations in roots and tea shoots, *TF* of N and Zn showed different changing patterns in response to Zn supply (**Figures 2C, F**). However, both N and Zn *TFs* were significantly reduced after Zn supply at both moderate and high N levels.

### 3.3 Soil physicochemical properties

Soil physicochemical properties in the rhizosphere of tea plants varied significantly among fertilizer treatments (**Table 1**). At moderate N level, a 2.70% decrease of pH value, a 40.6% increase of organic matter (OM) content, a 40.6% increase of MBC, a 36.1% increase of MBN and a 20.9% decrease of available potassium (AK) content were determined in the rhizosphere soil of tea plants after Zn supply. However, the changes in pH value and MBC at high N level showed different patterns. Zn addition altered the soil total nitrogen (TN) content at both moderate and high N levels with a 4.94% and 10.0% increase, respectively. Although the statistical analysis for NH_4_^+^-N and NO_3_^−^-N did not reach the significant level, the NH_4_^+^-N concentrations in the rhizosphere soil of tea plants after Zn supply at both moderate N and high N levels increased 1.08- and 1.04-folds, respectively, while the NO_3_^−^-N concentrations decreased to 3.38% and 6.38%, respectively. However, the Zn addition significantly elevated the concentrations of total Zn and DTPA-Zn in the rhizosphere soil of tea plants at both moderate N (102% and 47.2%) and high N (48.4% and 112%) levels. Apart from S-PPO, the activities of the other soil enzymes were significantly affected by exogenous fertilization. At moderate N level, a 21.6% increase of S-UE, a 90.8% increase of S-SC and a 18.2% increase of S-POD were found in the rhizosphere soil of tea plants after Zn supply, while these increment values reached into 27.2%, 107% and 24.8% at high N level, respectively.

**Table 1:** The physicochemical properties of rhizosphere soil of tea plants in response to different combinations of N and Zn fertilization.

Soil physicochemical properties	CK	Zn	N	N+Zn
pH (1:2.5)	5.70 ± 0.020a	5.55 ± 0.015b	5.15 ± 0.023d	5.30 ± 0.018c
Organic matter (g/kg)	3.58 ± 0.111b	5.03 ± 0.085a	4.93 ± 0.232a	5.28 ± 0.138a
MBC (mg/kg)	70.4 ± 1.96c	102 ± 0.869a	107 ± 2.34a	92.7 ± 2.79b
Total N (g/kg)	0.733 ± 0.014a	0.733 ± 0.009a	0.713 ± 0.023a	0.695 ± 0.023a
MBN (mg/kg)	21.0 ± 1.91c	28.6 ± 1.14b	30.7 ± 0.827b	35.9 ± 1.05a
Available P (mg/kg)	58.2 ± 1.96a	57.9 ± 1.66a	37.4 ± 0.997b	19.4 ± 0.951c
Available K (mg/kg)	89.5 ± 2.25a	70.8 ± 3.15b	89.3 ± 1.70a	60.0 ± 2.27c
NO_3_^-^-N (mg/kg)	9.16 ± 0.388b	8.85 ± 0.282b	14.3 ± 1.20a	13.4 ± 0.920a
NH_4_^+^-N (mg/kg)	31.4 ± 0.572b	34.0 ± 0.986b	42.2 ± 1.68a	44.1 ± 0.869a
Total Zn (mg/kg)	56.4 ± 4.09c	114 ± 4.41a	62.8 ± 1.08c	93.1 ± 1.89b
DTPA-Zn (mg/kg)	0.930 ± 0.092c	5.33 ± 0.345a	1.44 ± 0.204c	3.06 ± 0.385b
S-UE (mg/g.24h)	0.091 ± 0.003c	0.111 ± 0.002c	0.143 ± 0.005b	0.182 ± 0.016a
S-SC (mg/g.24h)	2.45 ± 0.164d	4.67 ± 0.061b	3.30 ± 0.154c	6.83 ± 0.359a
S-POD (mg/g.24h)	10.6 ± 0.589c	12.5 ± 0.234b	12.1 ± 0.471bc	15.1 ± 0.631a
S-PPO (mg/g.24h)	3.65 ± 0.117a	4.01 ± 0.166a	3.58 ± 0.123a	3.90 ± 0.146a

Data represent the means ± standard errors (n = 4). Different letters in the same row indicate significant differences among all treatments at *P* < 0.05. CK, control; Zn, Zn treatment; N, N treatment; N+Zn, N+Zn treatment.

### 3.4 Soil microbial diversity and composition

**Figure 3:**
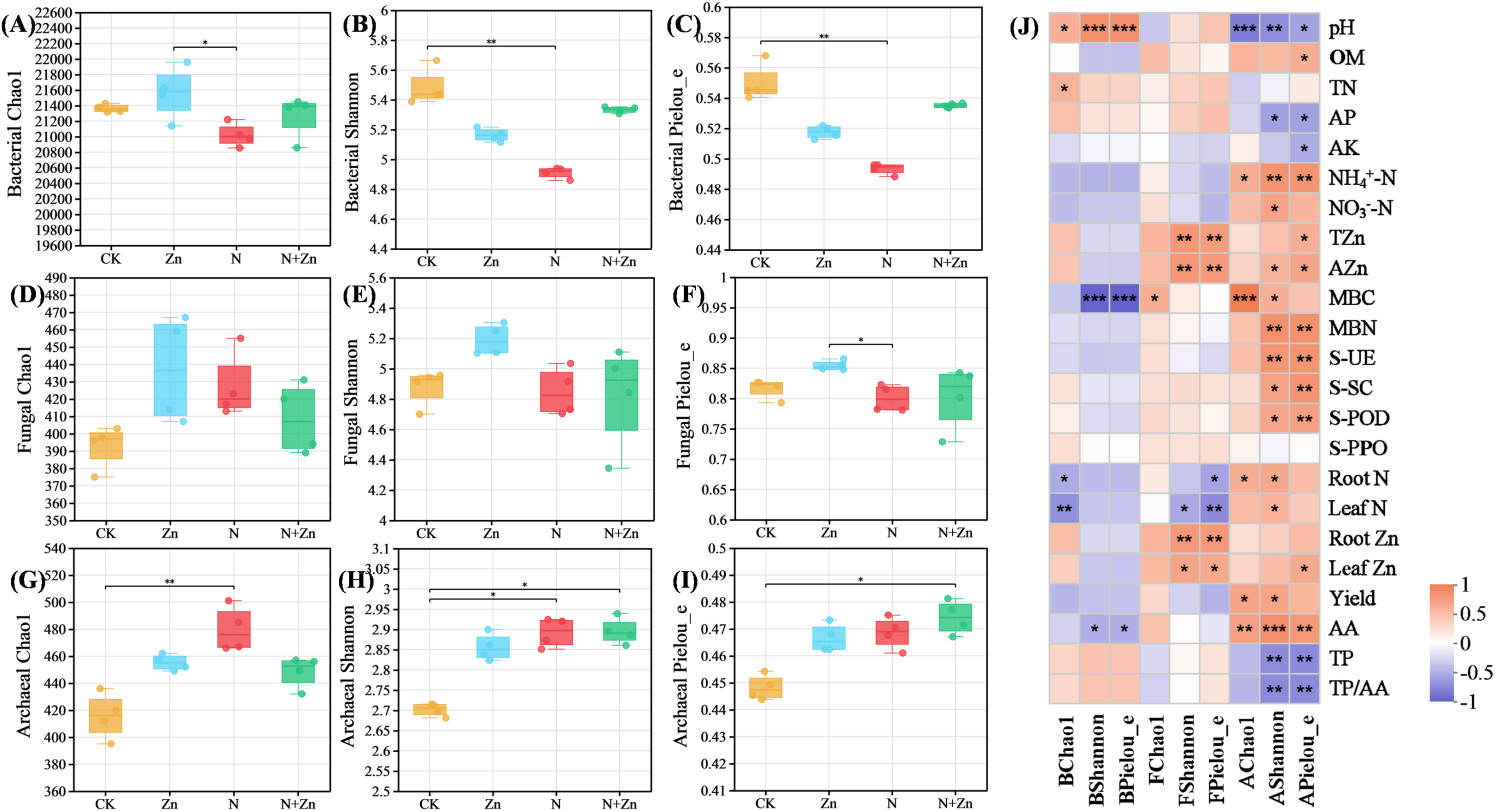
Box plots of α-diversity indices of bacteria **(A–C)**, fungi **(D–F)** and archaea **(G–I)** in response to different combinations of N and Zn fertilization based on Kruskal-Wallis H test. **(J)** Spearman correlation between soil properties, enzyme activities and microbial α-diversity indices. * indicates significance at *P* < 0.05. ** indicates significance at *P* < 0.01. *** indicates significance at *P* < 0.001.

**Figure 4:**
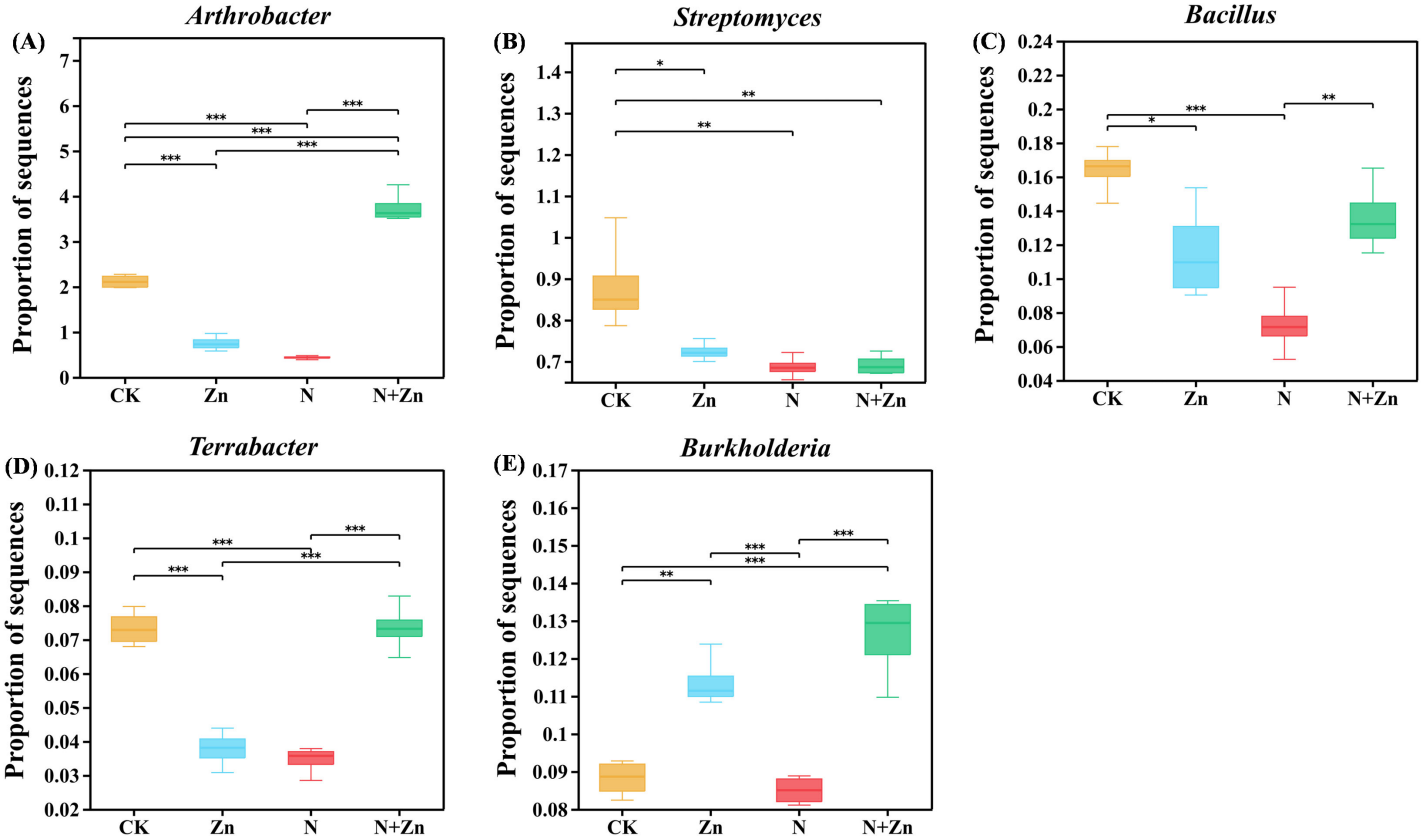
Box plots of relative abundance of genera *Arthrobacter* **(A)**, *Streptomyces* **(B)**, *Bacillus* **(C)**, *Terrabacter* **(D)**, and *Burkholderia* **(E)** in response to different combinations of N and Zn fertilization based on Kruskal-Wallis H test. * indicates significance at *P* < 0.05. ** indicates significance at *P* < 0.01. *** indicates significance at *P* < 0.001.

Soil metagenome sequencing was used to reveal the effect of different combinations of N and Zn fertilization on soil microbial diversity and community. At moderate N level, Zn supply increased bacterial, fungal and archaeal Chao1 indexes, but decreased the fungal and archaeal Chao1 indexes at high N level (**Figures 3A, D, G**). The abundance and evenness of the soil bacterial community were decreased after Zn addition at moderate N level, while there were increases at high N level (**Figures 3B, C**). However, at moderate N level, Zn supply increased the Shannon and Pielou_e indexes of both fungi and archaea, while the increments were lessened after Zn application at high N level (**Figures 3E, F, H, I**). Spearman correlation analysis indicated that microbial alpha diversity indexes were significantly changed with soil properties and enzyme activities, especially the archaeal Shannon index and archaeal Pielou_e index (**Figure 3J**). Variations in the microbial community across four treatments were evaluated by the non-metric multidimensional scaling (NMDS) analysis and PERMANOVA analysis. NMDS analysis based on Bray-Curtis dissimilarities showed that the bacterial microbiota, fungal microbiota and archaeal microbiota formed four distinct clusters based on the fertilization treatment (*P* < 0.01) respectively (**Supplementary Figures 1A–C**), which is consistent with the results of PERMANOVA analysis based on Bray-Curtis (**Supplementary Table 1**). At phylum level, Zn application reduced the RAs of *Actinomycetota*, *Pseudomonadota*, *Bacteroidota* and *Nitrospirota* at moderate N level, but increased the RAs of *Actinomycetota*, *Bacteroidota* and *Verrucomicrobiota* at high N level (**Supplementary Figure 2**). At genus level, Zn supply significantly decreased the RAs of *Arthrobacter*, *Streptomyces*, *Bacillus* and *Terrabacter* at moderate N level, but obviously increased the RAs of *Arthrobacter*, *Bacillus*, *Terrabacter* and *Burkholderia* at high N level (**Figure 4**). The results of the VIF analysis (VIFs < 20) and RDA analysis suggested that the OM, NH_4_^+^-N, NO_3_^−^-N, AZn, and MBC contributed to the variations in the soil microbial community structure among treatments (**Supplementary Figure 3**).

### 3.5 Co-occurrence networks of soil microbial communities

**Figure 5:**
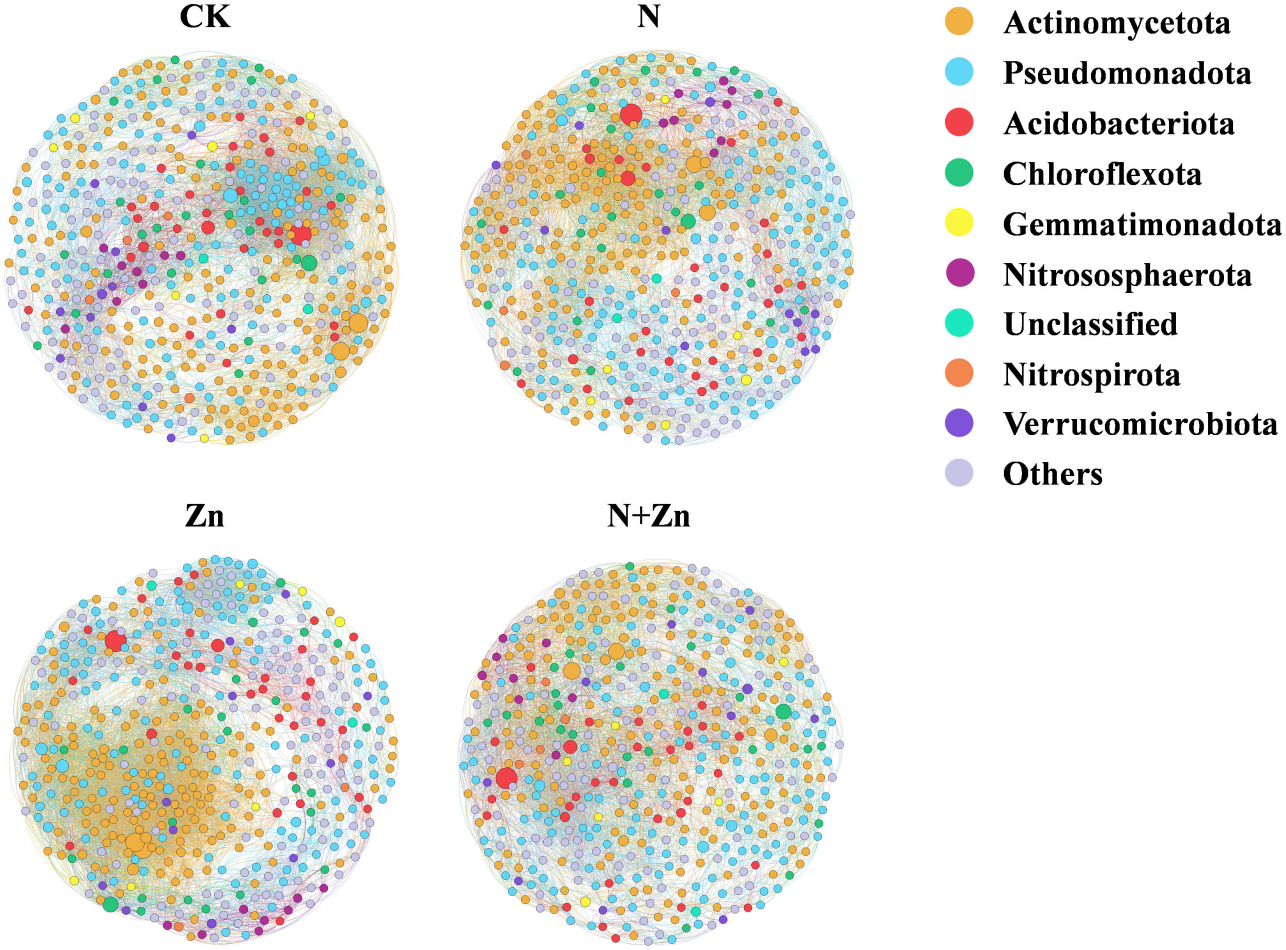
Network of co-occurring bacterial, fungal and archaeal genera under different combinations of N and Zn fertilization. Only Pearson’s correlation coefficient (|r| > 0.6 and *P* < 0.05) is shown.

NST analysis showed that NST values in CK and Zn treatments were 58.4% and 75.9%, respectively, higher than the threshold (50%). While, the NST values in N and N+Zn treatments were lower than 50%, with the values of 32.3% and 43.9%, respectively (**Supplementary Figure 4**). Microbial network analysis showed that the network nodes were mainly composed of the phyla *Actinomycetota* and *Pseudomonadota*, accounting for the majority of microbial interactions (> 50%) (**Figure 5**). The rate of positive associations was higher after Zn supply at moderate N level than under the other treatments. N addition decreased the edge numbers, average degree and graph density at both moderate and high Zn levels. While, at both moderate and high N levels, these above parameters were higher after Zn supply (**Table 2**). Nodes with higher within-module connectivity (Zi > 2.5) or higher among-module connectivity (Pi > 0.62) were identified as key taxa that can function through the inter-module exchange of energy and matter. Spearman correlations between the RAs of the key taxa and soil environmental factors were explored based on spearman analysis (**Supplementary Figures 6**–**8**). Results indicated that most of the key taxa under CK treatment, such as *Pseudomonadota* and *Actinomycetes*, were positively correlated with soil pH, AP, AK, TP and TP/AA, but negatively correlated with soil OM, NH_4_^+^-N, NO_3_^−^-N, MBC, MBN, S-UE, S-SC, S-POD, root N, leaf N, root Zn, leaf Zn, yield and AA, while *Acidobacteriota* and *Chloroflexota* showed contrast correlations (**Supplementary Figure 6A**). *Ktedonobacteria*, *Verrucomicrobiae* and *Thermoplasmata*, the primary key taxa under Zn treatment, showed positive correlations to soil OM, TZn, AZn, MBC, MBN, S-UE, S-SC, S-POD, S-PPO, root Zn, leaf Zn and AA but negative correlations to soil pH, TP, TP/AA (**Supplementary Figure 6B**). The dominating key taxa under N treatment, such as *Burkholderiales*, *Rhodocyclales*, *Hyphomicrobia*les, *Bacteroidales* and *Sphingobacteriales* were positively correlated to soil pH, AP, AK, TP and TP/AA, but negatively correlated to OM, NH_4_^+^-N, NO_3_^−^-N, TZn, AZn, MBC, MBN, S-UE, S-SC, S-POD, leaf Zn, yield and AA (**Supplementary Figure 7A**). As comparison, the primary key taxa after N-Zn co-fertilization, like *Caulobacterales*, *Burkholderiales*, *Sphingomonadales*, *Micrococcales* and *Micromonosporales*, showed positive correlations to soil pH, AP, TP and TP/AA but negative correlations to soil OM, NH_4_^+^-N, TZn, AZn, MBC, MBN, S-UE, S-SC, root Zn, leaf Zn, yield and AA (**Supplementary Figure 7B**).

**Table 2:** The microbial co-occurrence network topological properties in response to different combinations of N and Zn fertilization.

Topological properties	CK	Zn	N	N+Zn
Total nodes	500	500	500	500
Total links	8267	10062	7392	7576
Positive correlation rations	62.96%	83.17%	64.45%	58.45%
Average degree (avgk)	33.1	40.2	29.6	30.3
Density	0.066	0.081	0.059	0.061
Modularity	0.623	0.542	0.629	0.612
Average clustering coefficient (avgCC)	0.618	0.636	0.596	0.604
Average path distance (GD)	3.96	3.83	3.96	3.95

CK, control; Zn, Zn treatment; N, N treatment; N+Zn, N+Zn treatment.

### 3.6 Soil microbial functional gene profiles involved in N cycling and Zn resistance

**Figure 6:**
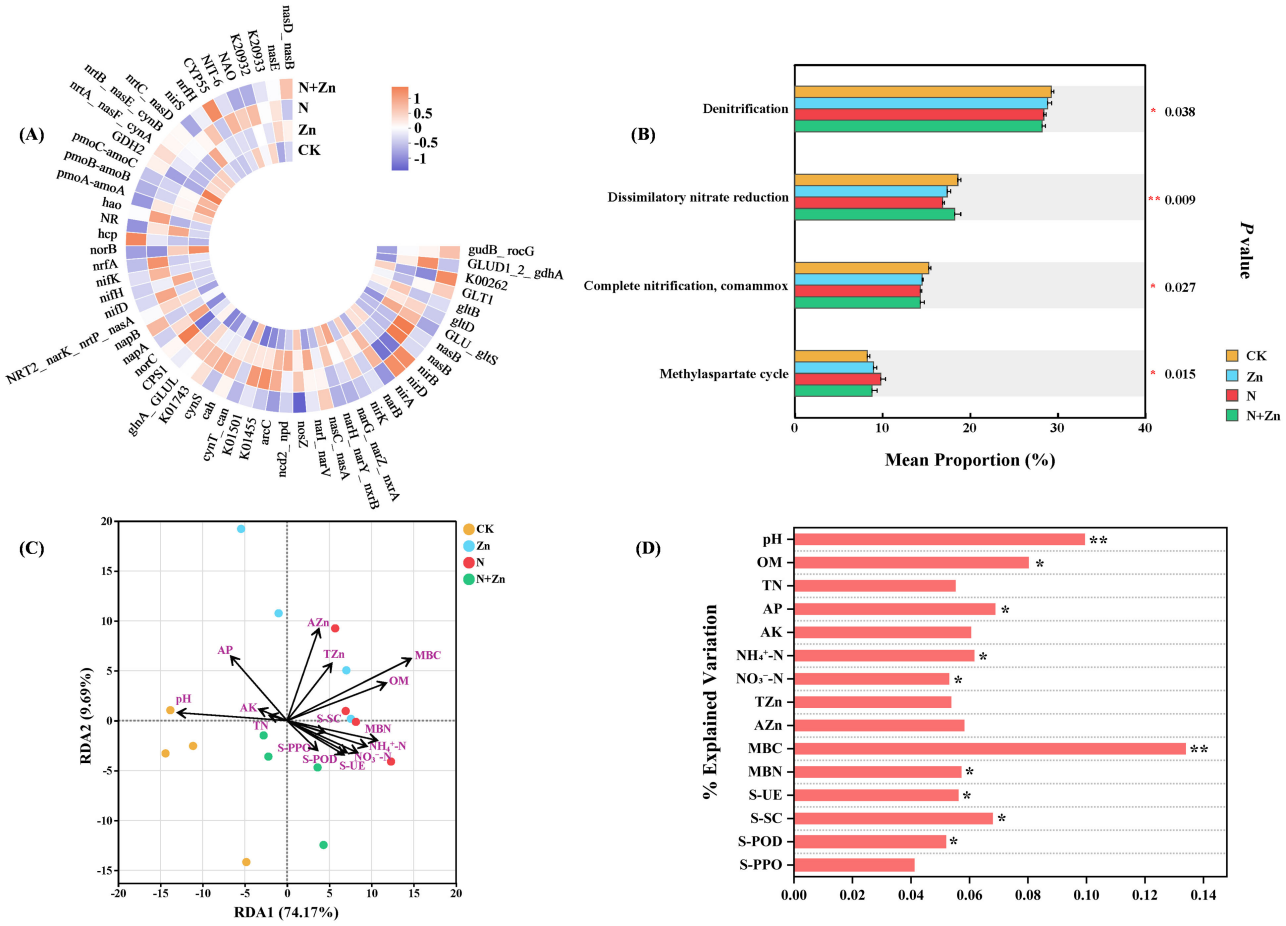
Heatmaps displaying relative abundances of genes related to N cycling **(A)**. Genetic potential pathways of soil N cycling in response to different combinations of N and Zn fertilization based on Kruskal-Wallis H test. **(B)**. RDA showing the relationships of environmental parameters with N cycling genes **(C)**. Hierarchical partitioning analysis and significant testing were performed to evaluate the explanatory variance and significance of various explanatory variables for genes related to N cycling **(D)**. * indicates significance at *P* < 0.05. ** indicates significance at *P* < 0.01.

**Figure 7:**
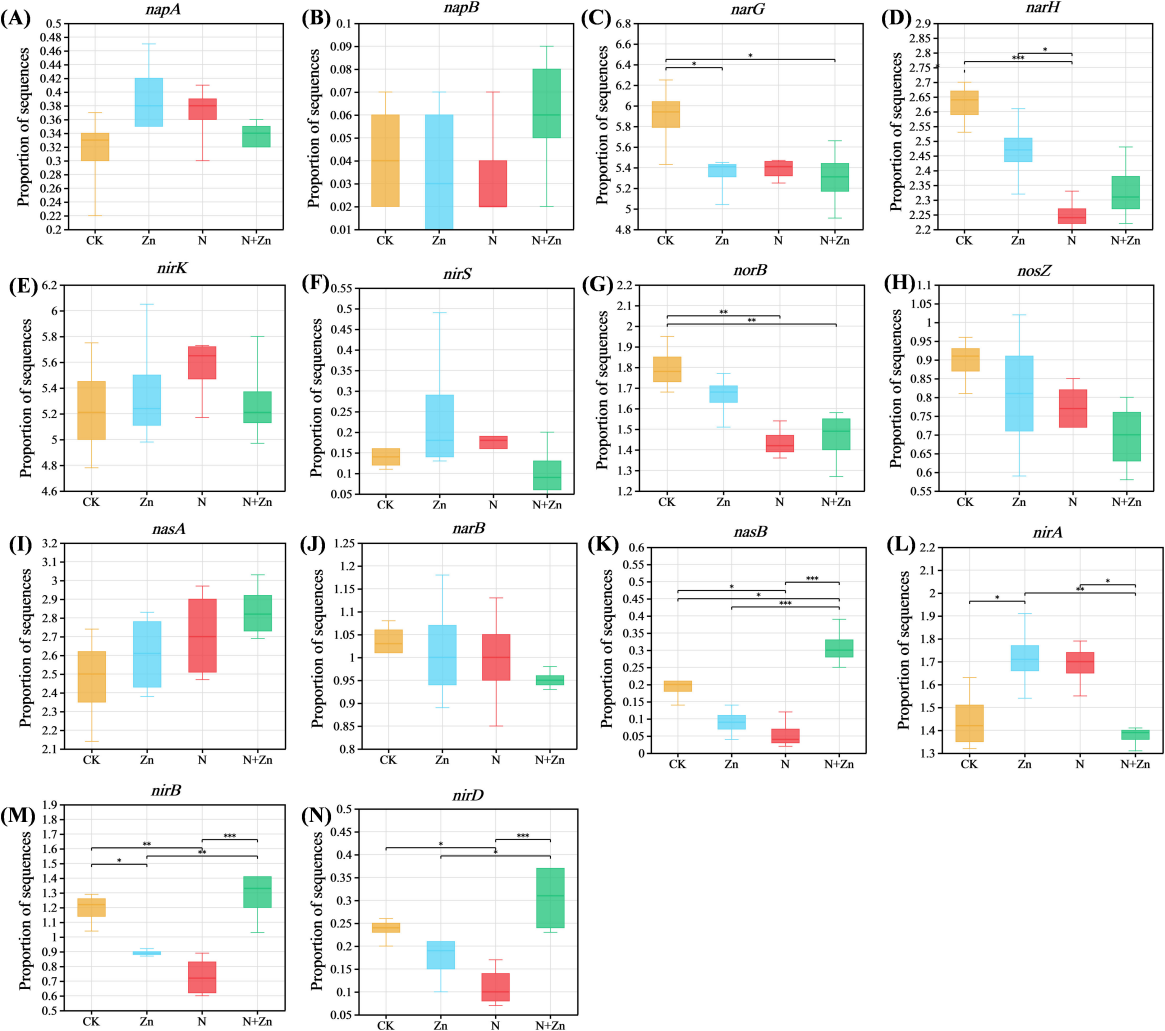
Box plots of denitrification **(A–H)**, assimilatory nitrate reduction **(I–L)** and dissimilatory nitrate reduction **(A–D, M, N)** functional genes in response to different combinations of N and Zn fertilization based on Kruskal-Wallis H test. * indicates significance at *P* < 0.05. ** indicates significance at *P* < 0.01. *** indicates significance at *P* < 0.001.

Functional changes of soil bacterial communities in different fertilization treatments were investigated, specifically focusing on functions related to N cycling and Zn-resistance. There were 57 functional genes identified to be involved in metabolic potential pathways of soil N cycling (**Figure 6A**), such as N fixation, nitrification, denitrification, ammonia oxidation, and both assimilatory and dissimilatory nitrate reduction. It is noted that denitrification predominates, with dissimilatory nitrate reduction, and complete nitrification as the following pathway (**Figure 6B**). Denitrification genes *narG*, *nirK*, *narH*, *norB* and *nosZ* were found to be most abundant (**Figures 7C, D, E, G, H**), followed by *nirS*, *napA* and *napB* (**Figures 7A, B, F**). At moderate N level, Zn addition decreased the RAs of *narG, narH, norB* and *nosZ* (**Figures 7C, D, G, H**). Although the Zn application significantly decreased the RAs of dissimilatory nitrate reduction-related genes (*narG, narH, nirB, nirD*) at moderate N level (**Figures 7C, D, M, N**), it clearly increased the RAs of *narH, nirB* and *nirD* at high N level (**Figures 7D, M, N**). Within assimilatory nitrate reduction module, *nasA* genes predominate, succeeded by *nirA, narB* and *nasB*, all of which did not display regular fluctuations after fertilizations (**Figures 7I, J, K, L**). The RAs of nitrification-related genes (*hao*, *pmoA-amoA*, *pmoB-amoB*, *pmoC-amoC*) were found to be lower level and also displayed irregular fluctuations in response to fertilizations (**Supplementary Figure 9**), while for the complete nitrification module, there were significant reductions after Zn supply at both moderate and high N levels (**Figure 6B**). A total of 54 functionally distinct genes in the metagenomic data sets were predicted to be involved in metabolic potential pathways of Zn resistance, such as Zn regulation, Zn uptake, Zn efflux and Zn sequestration, and exhibited varying degrees of change (**Supplementary Figures 10A, B**). Within the metabolic process of Zn regulation, genes *zraR*/*hydH*, *zraS*/*hydG*, *irlR* and *actS* were found to be the most abundant. At moderate N level, Zn supply increased the RAs of *irlR* and *actS*. Although the Zn application increased the RAs of *irlR* and *actS* at high N level, it clearly down-regulated *zraR*/*hydH* and *zraS*/*hydG* meanwhile (**Supplementary Figure 11**). The RAs of Zn uptake-related genes (such as *znuC*/*yebM*, *pitA*, *fpvA*, *troB*, *zipB*, *mdrL*/*yfmO* and *troD*) displayed consistent trend after Zn fertilization at both moderate and high N levels. Zn addition decreased the RAs of Zn uptake-related genes at moderate N level, while up-regulated these above genes at high N level (**Supplementary Figure 12**). Also, genes *mdtB*, *mdtC*, *mdtA*, *czcC* and *czrB* participated in Zn efflux pump, were up-regulated by Zn supply at moderate Zn level. However, the levels of the above genes displayed downward trend after Zn addition at high N level (**Supplementary Figure 13**).

### 3.7 Correlations between soil properties, soil microbial functional gene profiles and tea characteristics

To explore the effects of environmental factors on microbial functional gene profiles, RDA was conducted on 15 environmental variables. Results revealed that soil properties explained 83.86% of the variation in N cycling genes (**Figure 6C**), along with 65.96% of the variation in Zn resistance genes (**Supplementary Figure 10C**). The results demonstrated that soil properties significantly correlated with microbial gene profiles after fertilization. Further, hierarchical partitioning analysis and significant testing were performed to evaluate the explanatory variance and significance of various explanatory variables for genes related to N cycling and Zn resistance, respectively. The analyses indicated that genes related to N cycling were mainly significantly influenced by MBC, pH and OM (**Figure 6D**). Genes involved in the Zn resistance were mainly influenced by MBC, pH and AZn (**Supplementary Figure 10D**). Surprisingly, spearman heatmaps demonstrated that most N cycling genes and Zn resistance genes were positively correlated with pH, AP, AK, TP, and TP/AA. And they exhibited an inverse relationship with the corresponding soil nutrient levels (**Supplementary Figure 15**).

**Figure 8:**
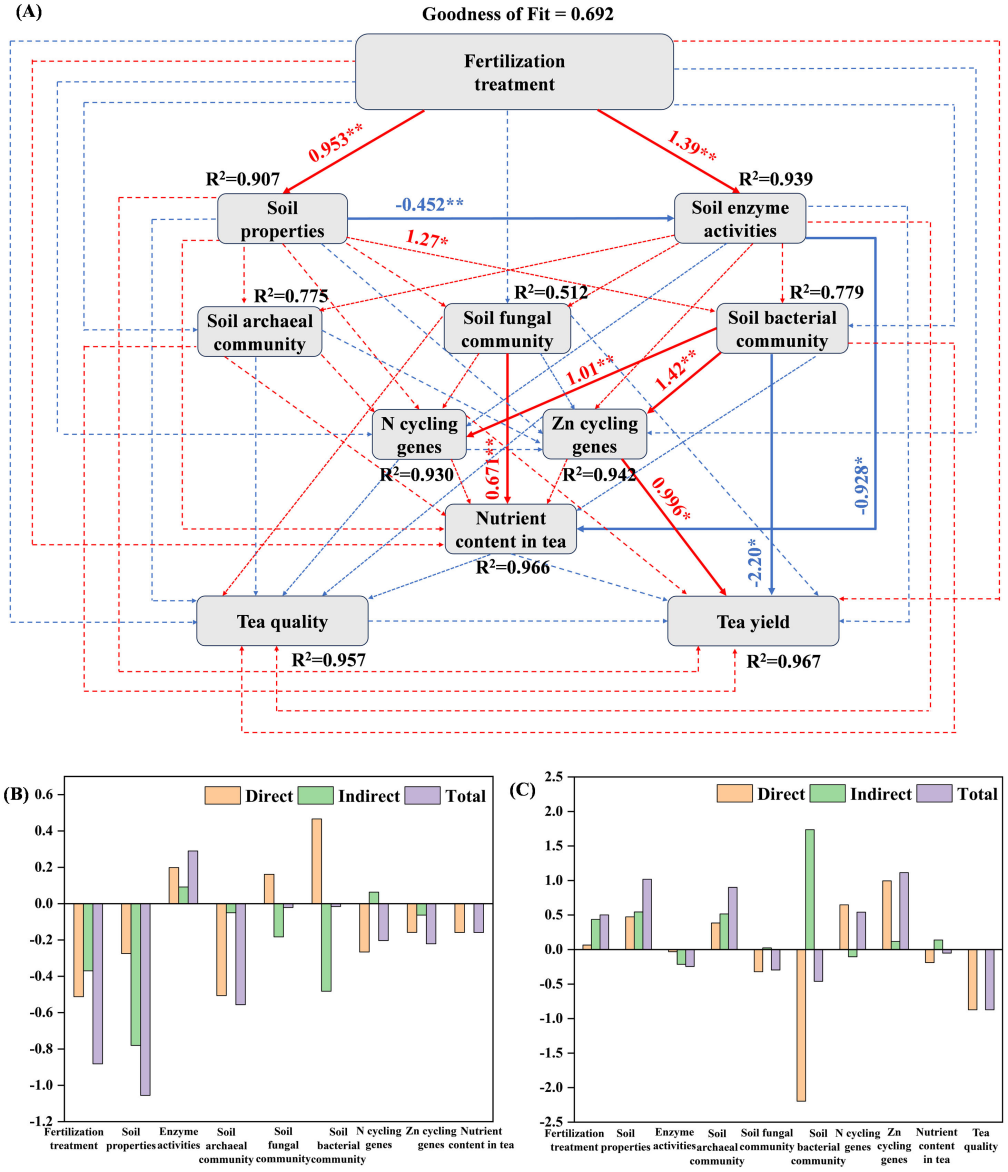
PLS-PM analysis showed the relationship between fertilization treatments, soil properties, soil enzyme activities, microbial community, N cycling/Zn resistance genes, nutrient content of tea, tea quality, and tea yield (goodness of fit = 0.692) **(A)**. Observed or latent variables are illustrated in the box. 1000 bootstraps were conducted to calculate path coefficients. The red and blue lines represent positive and negative effects, respectively. Solid and dashed lines indicate significant and non-significant effects, respectively. The number on the line represents the total effect value (* indicates significance at *P* < 0.05. ** indicates significance at *P* < 0.01.). Direct and indirect effects of the variables on the quality **(B)** and yield **(C)** based on the PLS-PM analysis.

**Figure 9:**
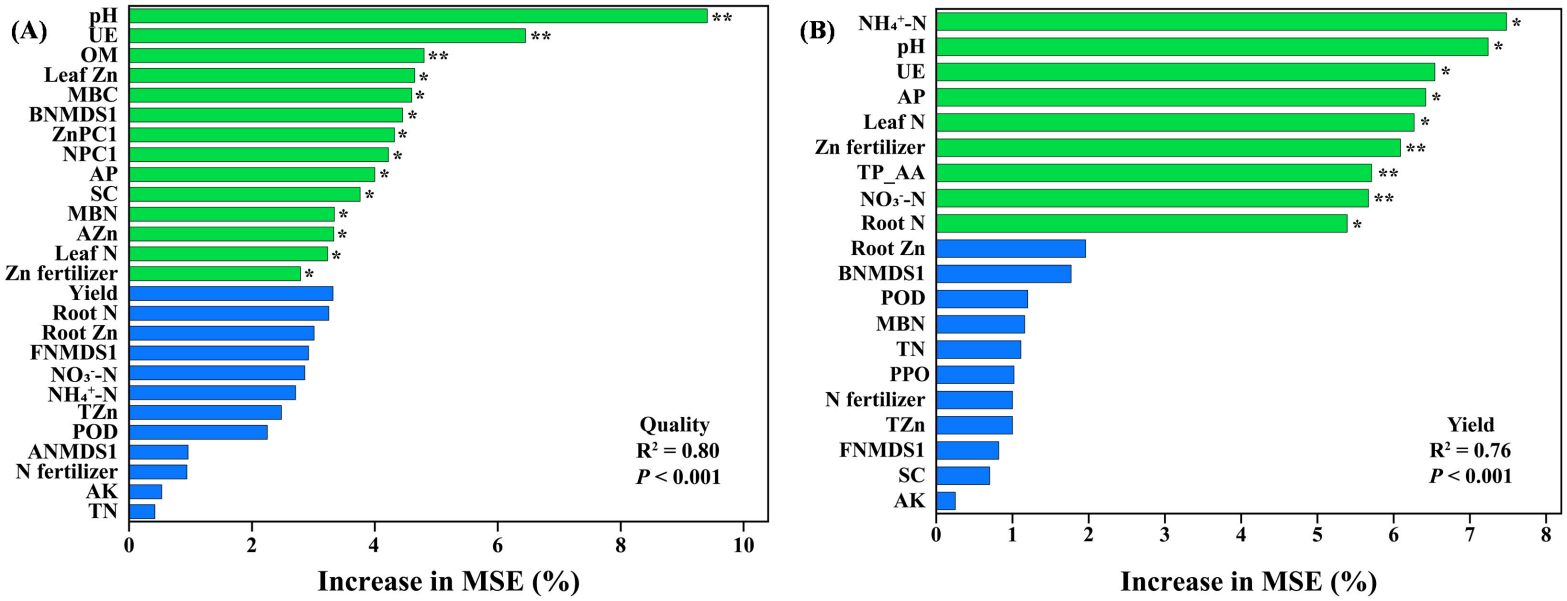
Random forest analysis for the determination of the factors affecting the tea quality **(A)** and yield **(B)**. * indicates significance at *P* < 0.05. ** indicates significance at *P* < 0.01.

Partial least squares path models (PLS-PM) were constructed to determine the relationships among fertilization treatments, soil properties, soil enzyme activities, microbial community, N cycling/Zn resistance gene profiles, nutrient content of tea, tea quality, and tea yield (**Figure 8A**). The results indicated that fertilization treatment had direct negative effects on tea quality and yield with non-significant difference. Standardized effect analysis revealed that tea quality was most strongly influenced by soil properties (total effect values = 1.06) and fertilization practices (total effect values = 0.882) among all variables examined (**Figure 8B**). With regard to tea yield, Zn-resistance functional gene profiles showed the highest total effect (1.11), followed by soil properties (1.02) and archaeal communities (0.901) (**Figure 8C**). Thus, fertilization indirectly affected the microbial community, N cycling/Zn resistance gene profiles and nutrient content in tea, by directly altering the soil properties and soil enzyme activities significantly. In summary, the PLS-PM results demonstrated that fertilization treatment altered tea quality and yield by influencing soil properties, soil enzyme activities, microbial community, N cycling/Zn resistance gene profiles, and ultimately affecting the nutrient content in tea. Further, random forest analysis was applied to predict the key influential factors of tea yield and quality (**Figure 9**). The results suggested that the variation in tea quality was mainly affected by pH, S-UE, OM, leaf Zn, MBC, BNMDS1, ZnPC1, NPC1, AP, S-SC, MBN, AZn, leaf N, Zn fertilizer (**Figure 9A**). The most correlated factor for tea quality was soil pH. Based on the random forest analysis, the soil NH_4_^+^-N content, pH, S-UE, AP, leaf N, Zn fertilizer, TP/AA, NO_3_^−^-N, root N were the main factors affecting tea yield (**Figure 9B**). In addition, tea yield was most associated with soil NH_4_^+^-N content and pH.

## 4 Discussion

### 4.1 Alterations of tea yield, quality and nutrient contents in tea leaves in response to different combinations of N and Zn fertilization

N and Zn are essential nutrient elements for tea growth, playing vital roles in metabolite synthesis. Previous studies have reported synergistic interactions between Zn and N in plants. For example, Kutman et al. (2010) have reported that N is important for the acquisition and grain allocation of Zn in wheat. Synergistically, applying Zn can positively affect the N acquisition, root-to-shoot translocation, and preferential distribution, as well as the plant biomass and yield production in rice, barley and maize (Almendros et al., 2019; Montoya et al., 2021; Ji et al., 2022). Consistent results have been showed in the present study that Zn supply significantly increased tea quality by contributing to the synthesis of AA and decreasing the TP content at moderate N level. This might be ascribed to the reason that Zn could participate in the regulation of sugar conversion and the formation of quality components in tea plants, such as amino acids, catechins and aroma substances (Zhang et al., 2017). In addition, the difference in the tea yield among different treatments indicated that N fertilizer contributed the most to the increase in tea production compared with Zn application. This is in agreement with the result of a previous meta-analysis, which revealed that application of synthetic N fertilizer increased tea production by nearly 70% (Qiao et al., 2018). While, the inconspicuous effect of Zn addition on tea yield might be due to the reason that soil available Zn was adequate to supply the low requirements of the tea plants. In contrast to the reported results, in the present study, Zn application did not significantly increase the N uptake in tea plants, especially in roots and tea shoots. This phenomenon might be explained by the following two reasons: firstly, Zn supply could facilitate the vegetative growth of tea plants, such as new tea shoots and root system. Although the total N accumulation of the tea plants increases, the N is rapidly diluted into a larger biomass (more leaves, longer root systems), resulting in a relative decrease in N concentration per unit mass in root or tea shoots; secondly, as the key activator for many enzymes, adequate Zn could accelerate the N assimilation by promoting the transformation of inorganic N (NO_3_^−^-N and NH_4_^+^-N) into amino acids in plant tissues, thereby leading to the reduction of the transient accumulation of inorganic N in roots and tea shoots. These results indicated that N and Zn should be combined optimally to achieve high-yield and high-quality tea.

### 4.2 Changes of soil N availability in response to different combinations of N and Zn fertilization

NO_3_^−^-N and NH_4_^+^-N are predominant forms of inorganic N in soil, both of which can be absorbed by plants directly. However, tea plant is more likely to uptake NH_4_^+^ from the soil of tea plantations (Ruan et al., 2007). In the present study, we found Zn supply increased the concentrations of soil TN and NH_4_^+^-N, but decreased the concentration of NO_3_^−^-N at both moderate and high N levels. This phenomenon might be finally put down to the positive influence on urea hydrolysis and nitrate reduction after Zn addition, leading to the accumulation of NH_4_^+^-N and the reduction of NO_3_^−^-N. Consistent with the above deduction, soil metagenomic analysis showed that Zn application promoted nitrate reduction by up-regulating the RAs of nitrate reductase genes (*narH*, *nasA*, *nasB*, *napB*), nitrite reductase genes (*nirB*, *nirD*, *nasB*), and nitric oxide reductase genes (*norB*) at high N level. Also, positive correlations were observed between soil MBN, S-UE activities and NH_4_^+^-N content (**Supplementary Table 2**). In conclusion, Zn application at high N level could promote the N conversion into NH_4_^+^-N and improve the N availability in the rhizosphere soil of tea plants, which is in line with Lv et al. (2022) who reported the similar results that Zn-N co-fertilization caused higher NH_4_^+^-N accumulations in rice rhizosphere soil. Therefore, optimal co-fertilization of Zn and N could increase soil N availability, and then facilitate the growth and nutrient acquisition in tea plants.

### 4.3 Modifications of soil microbial communities in response to different combinations of N and Zn fertilization

Microbial communities and abundances are the critical factors driving soil nutrient cycle and contributing to the maintenance of soil functions (Jiao et al., 2021; Morugán-Coronado et al., 2022; Trivedi et al., 2020; Zheng et al., 2019). Previous studies have reported that the application of chemical fertilizers can alter the diversity and community structure of rhizosphere soil microorganisms in tea plantation (Ma et al., 2021; Tang et al., 2023; Yu et al., 2025). Our results revealed a distinct hierarchical response among microbial domains that archaeal diversity was more sensitive to N-Zn fertilization than bacterial or fungal diversity. This could be explained by the niche differentiation between archaea, bacteria and fungi in relation to their different responses to changes in soil characteristics induced by fertilization (Harris, 2009), which is consistent with the results of correlation analysis that the archaeal Shannon index and archaeal Pielou_e index were significantly changed with soil properties and enzyme activities. The increases in bacterial Chao 1 indexes after Zn application at both moderate and high N levels, which is regarded as accounting for species richness and reflecting rare species change, suggesting that Zn application might be able to recruit more heavy metal-tolerant bacteria in the rhizosphere soil of tea plants. However, heavy metal-tolerant fungi and archaea might not be able to live in the rhizosphere soil after Zn application at high N level.

Variations were also observed in both the structure and composition of the soil rhizobacterial community under different combinations of N and Zn fertilization. Contrary to the oligotroph-copiotroph paradigm (Fierer et al., 2007; Yao et al., 2017), N fertilization increased the RAs of typically oligotrophic *Acidobacteriota* and *Chloroflexota*, while reducing the RAs of copiotrophic *Pseudomonadota*. This inversion is best explained by fertilization-induced soil acidification, which directly inhibits acid-sensitive copiotrophs, and then create vacant niches for acid-tolerant oligotrophs to occupy. Furthermore, the conditional response of denitrifying genera (e.g., *Arthrobacter*, *Bacillus* and *Terrabacter*) to Zn, which were suppressed at moderate N level but stimulated at high N level, highlights how the ecological function of a microbial group can be reversed by N availability. This showcased a complex interaction between nutrient amendment and metal ion effects. *Chloroflexi* and *Acidobacteria*, known for their Zn stress tolerance and ability to produce organic acids during organic matter decomposition (Lv et al., 2022), showed a positive correlation with total Zn and AZn. However, under N+Zn treatment, the decline in *Acidobacteria* and *Chloroflexi* may reflect the inhibitory effect of excess Zn on these groups in an acidic rhizosphere environment. Although stochastic processes dominated the assembly processes of microbial communities at moderate N level, N addition significantly decreased the NST value, indicating that the deterministic process ratio increased with increasing N. Soil acidification induced by N addition might be one of the key drivers of deterministic processes in microbial communities (Liu et al., 2021).

Previous studies have shown that associations in co-occurrence networks could imply interactions among microorganisms or a shared ecological niche (Guo et al., 2024). In the present study, Zn addition not only increased network complexity as indicated by the higher edge numbers, average degree and graph density, but also improved network robustness with larger area of robustness curve under random attack (**Supplementary Figure 5**) (Kajihara and Hynson, 2024). However, highest NST value under N+Zn treatment suggested that it was stochastic processes dominated the assembly processes of microbial communities rather than deterministic process like Zn stress. This situation might be ascribed to the greater influence of key taxa on network stability, which could collapse quickly when targeted attack occurs (Tipton et al., 2018). In contrast, N fertilization simplified networks but improved the network modularity and average path distance. High modularity and low connectivity were considered to have the potential to limit disturbances to specific modules, thus preventing their spread across the entire network (Xiao et al., 2023). Under N-deficient conditions, microorganisms enhance cooperation with one another by enriching more marker genera to help their survival (Kang et al., 2024). Appropriate N supplement increased the competition between microorganisms and was more favorable for maintaining the stability of the microbial community network (Zhou et al., 2020). The abundance of key microbial taxa is crucial for the process of nutrient cycling and soil function (Nielsen et al., 2017). The current study underscored the association between the abundance of key taxa and soil properties, tea characteristics. Some of these microbial taxa (i.e. *Cupriavidus*, *Usitatibacter*, *Nitrobacter*, *Rhizobacter*, and *unclassified_f__Candidatus_Binatellaceae*) have been reported to promote plant growth by N fixation and solubilizing nutrients such as P, produce various phytohormones and siderophores, and participate in organic matters degradation and resistance to heavy metal stress (Ducret et al., 2021). While it is true that a correlation does not necessarily imply causation, our results revealed notable associations between the abundances of several key genera and specific plant nutrients. For instance, *Cupriavidus*, key microbial taxa of both Zn and N+Zn treatments, exhibited positive correlations with soil TZn and AZn concentrations, which has been reported to participate in Zn resistance and immobilization under heavy metal contaminated soils (Ducret et al., 2021). In addition, *unclassified_f__Candidatus_Binatellaceae* was positively associated with soil TN concentrations but negatively correlated with NO_3_^−^-N levels, a pattern supported by the presence of functional genes *narG*, *nirK* and *nirS* that mediate the reduction of NO_3_^−^ to NO_2_^−^, indicating its potentially important role in soil N cycling.

### 4.4 Response of soil microbial functional gene profiles involved in N cycling and Zn resistance under different combinations of N and Zn fertilization

Soil microorganisms serve as the pivotal biological agents driving the nutrient biogeochemical cycle, with research into their community structure and functional genes holding significant scientific importance for revealing soil nutrient metabolism processes (Chen et al., 2020). In the present study, soil microbial functional pathways related to nutrient cycling were significantly changed after fertilization, especially soil N cycling and response to Zn stress. Although biological N fixation is a crucial metabolic potential pathway of N acquisition, this study found a notably low level of N-fixing pathways and the lower RAs of functional genes, such as *nifH*. This may be caused by the N-rich soil environment at moderate and high N levels (Liu et al., 2025), evidenced by the increase of soil TN, NH_4_^+^-N contents as well as MBN concentrations. Both nitrification and complete nitrification are key metabolic potential pathways of N metabolism, which could oxidize NH_3_/NH_4_^+^ to NO_3_^−^. However, as compared to nitrification, complete nitrification pathway predominated in the present study, indicating that complete nitrification might play potentially important roles in the nitrifying process under the effect of comammox. Notably, the RAs of nitrate reductase genes (*narH*, *nasA*, *nasB*, *napB*), nitric oxide reductase genes (*norB*), and nitrite reductase genes (*nirB*, *nirD*) were increased after Zn supply at high N level. *narH*, which performs the first rate-limiting step during soil denitrification, is known as a major factor driving N turnover (Li et al., 2020). The differences in the RAs among these above genes may be associated with the RAs of denitrification and nitrate reduction pathways. Consequently, we speculated that Zn supply at high N level in tea plantation might be positively associated with soil denitrification and nitrate reduction pathways, evidenced by the decrease of NO_3_^−^-N levels and the increase of NH_4_^+^-N concentrations in the rhizosphere soil.

Previous researches have demonstrated that the different adaptation strategies imperative for survival of microbes under Zn stress include reducing the sensitivity through permeability barriers, enhancing efflux by transporters, enzymatic detoxification, reduction of metal ions and extracellular and intracellular sequestration (Ducret et al., 2021; Dinesh et al., 2023). Microorganisms manage Zn stress through primary and secondary filtration systems, such as P-type ATPase, chemiosmotic cation diffusion facilitators (CDF), and resistance-nodulation-division (RND) pumps (Raghavan et al., 2023). In the present study, at high N level, Zn supply decreased the RAs of some metabolic potential pathways, such as Zn regulation and Zn efflux, but promoted the RAs of Zn uptake pathway, supported by the significant reduction of the RAs of *zraR*/*hydH* and *zraS*/*hydG*, obvious down-regulations of *mdtB*, *mdtC* and *mdtA* as well as the distinct increase of the RAs of *znuC*/*yebM*, *pitA* and *troB*. This situation is inconsistent with the well-known adaption strategy for maintaining Zn homeostasis (Ducret et al., 2021; Raghavan et al., 2023), which could be ascribed by the significant effect of low pH in the rhizosphere environment after urea fertilization. On the one hand, pH plays a pivotal role in determining the dominance of bacteria or fungi, thereby altering the microbial community structure and ecosystem functionality (Zhu et al., 2025). On the other hand, pH can modulate microbial cell membrane permeability, affecting the absorption of Zn ions and the expression of intracellular resistance genes (Wang et al., 2023).

Overall, integrating soil microbial communities with fertilization management can provide valuable insights into the nutrient cycling and soil ecological functions, and help establish a connection between soil microbial communities and environmental changes. However, these conclusions are merely speculations on the basis of the results of read counts from assembly-based metagenomics datasets, which could not directly represent the absolute abundance of genes. Absolute quantification and independent validation (such as qPCR) will address this limitation in the future work.

### 4.5 Factors influencing tea yield and quality under different combinations of N and Zn fertilization

The results of the PLS-PM and random forest analyses showed that, at the abiotic level, soil properties such as pH, OM, AP and AZn were key factors influencing tea quality, while NH_4_^+^-N, pH, AP and NO_3_^–^-N were critical determinants of tea yield, which is consistent with the results of correlation analyses (**Supplementary Table 3**). Soil pH exerts a broad influence, from modulating nutrient bioavailability to directly steering leaf metabolic pathways toward amino acid or polyphenol dominance (Ruan et al., 2007). While N supply drives photosynthetic yield and the accumulation of nitrogenous quality compounds (Zhang et al., 2017), P underpins energy metabolism and growth architecture (Lin et al., 2012), and Zn fine-tunes enzyme activities critical for N assimilation and secondary metabolism (Upadhyaya et al., 2013). Acting as an integrative factor, OM enhances the physical and biochemical environment, supporting the processes driven by these nutrients. Within the biological compartment, bacterial communities, more responsive to fertilization than fungal communities, emerged as stronger predictors of plant outcomes. This aligns with their keystone role in decomposing OM and cycling nutrients like N and P, thereby functionally linking soil management practices (e.g., N-Zn co-fertilization) to nutrient availability for the plant (Saleem et al., 2019). The stability of fungal communities under perturbation may indicate different ecological roles. Thus, optimal yield and quality arise from fertilization strategies that simultaneously regulate key soil physicochemical properties and foster the microbial communities that sustain nutrient cycling and plant health.

## 5 Conclusion

A field experiment was conducted to systematically assess the effects of different combinations of N and Zn fertilization on the growth and nutrient uptake of tea plant, as well as on soil microbial communities and functional gene profiles in tea plantations. Results indicated that N fertilization contributed more substantially to the increment of tea yield than Zn fertilization, whereas Zn supplementation significantly improved tea quality. Moreover, at high N level, Zn application modulated rhizosphere microbial community, enhanced the RAs of genes related to denitrification and nitrate reductase, and increased the RAs of metabolic potential pathways of N metabolism, such as denitrification and dissimilatory nitrate reduction to ammonium. This shift might be associated with the decrease of NO_3_^−^-N levels and the increase of NH_4_^+^−N concentrations in the rhizosphere soil. PLS-PM models revealed that fertilization firstly altered soil physicochemical properties and enzyme activities, which in turn influenced rhizosphere bacterial community and functional gene profiles. These changes might contribute to nutrient accumulation in tea plants and ultimately affected tea yield. Random forest analysis further identified soil properties such as pH, OM, AP, NH_4_^+^−N, NO_3_^−^-N and AZn as the most influential factors associated with tea yield and quality. In summary, this study revealed the potentially important roles of optimized N-Zn co-fertilization in influencing tea yield and quality from a rhizosphere microbiological perspective, providing a scientific basis for refining integrated N-Zn management strategies in tea cultivation systems.

## Data Availability

The datasets presented in this study can be found in online repositories. The names of the repository/repositories and accession number(s) can be found below: https://www.ncbi.nlm.nih.gov/, PRJNA1395687.
