# Supplementary material for: Metagenomic insights into rhizosphere microbial communities and functional gene profiles associated with the responses of tea yield and quality to nitrogen-zinc co-fertilization
Source: Front Plant Sci. 2026 Jul 10;17:1852312. doi: 10.3389/fpls.2026.1852312 (PMC13395992; doi:10.3389/fpls.2026.1852312)
Supplement: Supplementary file 1 [file DataSheet1.pdf]

Supplementary Material

1 Supplementary Data

1.1 Analyses of AA and TP concentrations in tea shoots

The dried samples of tea shoots were extracted with boiling distilled water (ddH_2O) for 5 minutes to determine the AA contents (GB/T8314-2013). Briefly, 1 mL of the extracts were added into 0.5 mL of pH 8.0 phosphate buffer solution and 0.5 mL of 2% ninhydrin solution. Then heat the mixture in a boiling water bath for 15 min. After standing for 10 min, the absorbance was measured at 570 nm using a UV-VIS spectrophotometer in plastic cuvettes (Agilent Technologies, Cary Series 100 UV-Vis Spectrophotometer). Results were expressed as glutamic acid equivalent. The dried samples of tea shoots were extracted with 70 °C 70% MeOH for 10 minutes. The TP content in the extracts was determined following the Folin-Ciocalteau method using gallic acid as standard prepared in 70% MeOH to match the sample matrix (GB/T8313-2018). Briefly, 1 mL of the extracts were mixed with 5 mL Folin-Ciocalteau phenol reagent. After 5 min, 4 mL of sodium carbonate 7.5 % (w/w) was added to the mixture and incubated for 60 min in the dark at 25 °C. The absorbance was measured at 765 nm using a UV-VIS spectrophotometer in plastic cuvettes (Agilent Technologies, Cary Series 100 UV-Vis Spectrophotometer). Results were expressed as gallic acid equivalent.

1.2 Analyses of rhizosphere soil physicochemical properties and elemental concentrations

Soil pH values (soil:water = 1:2.5, m/m) were determined by calibrated pH meter (PB-10, Sartorius, Goettingen, Germany). Organic matter was measured according to the Walkley-Black wet digestion method. Total soil N was measured by Kjeldahl determination. Soil exch-NH_4^+-N and NO_3^--N were extracted using 2 M KCl and then measured with a SAN++ Flow Injection Analyzer (SKALAR Ltd, Netherlands). Soil available P was determined by the ascorbic acid-ammonium molybdenum method. Soil available K was extracted with ammonium acetate and determined by flame photometry. For soil Zn concentration analysis, soil samples were digested with HNO_3/$HClO_4$/HF (5:1:1, v/v/v). And the available Zn was extracted with diethylene triamine pentaacetic acid (DTPA) (0.005 mmol/L DTPA, 0.01 mmol/L $CaCl_2$, and 0.1 mmol/L triethanolamine, pH = 7.3). Soil samples were extracted with agent above in a 1:2 soil/solution mixture, then filtered after shaking for 2 h. Zn concentration in the digested solutions and the extracts were determined using an Inductively Coupled Plasma Mass Spectrometer (ICP-MS, PlasmaQuant®MS, Germany). Sample replicates, reagent blanks, soil and plant standard reference material (GBW07429 and GBW100351, the

National Research Center for Certified Reference Materials of China) were included in each batch of analysis to ensure the quality of analysis. The recovery of the standard for each element was 90-110%.

1.3 Statistical analysis

The random forest models were performed in R using "randomForest" and "rfPermute" packages, with the random seed set to 123 with otherwise default parameters (Liao et al., 2024). To optimize the parameters, the random forest model was initially trained on 70% of the data using the "randomForest" package. The remaining 30% of the data served as a validation set to assess the model's accuracy. After optimizing the parameters, the final model was constructed using all data based on following parameters: importance = TRUE, ntree = 500, and nrep = 1000. The significance of the models and cross-validated R^2 values were assessed based on 1000 permutations using all datasets with the "rfPermute" package in R. In the random forest model, a higher percentage of mean squared error (MSE) indicates a higher importance of a given factor (Jiao et al., 2018). The MSE for every decision tree with out-of-bag estimates based on random forest model was produced using "rfPermute" package, which assesses the relative importance of each predictor variable.

References:

Jiao, S. Chen, W.M., Wang, J.L., Du, N.N., Li, Q.P., Wei, G.H. Soil microbiomes with distinct assemblies through vertical soil profiles drive the cycling of multiple nutrients in reforested ecosystems. Microbiome 2018, 6, 146.

Liao, H.P., Liu, C., Zhou, S.G., Liu, C.Q., Eldridge, D.J., Ai, C.F., Wilhelm, S.W., Singh, B.K., Liang, X.L., Radosevich, M., Yang, Q.E., Tang, X., Wei, Z., Friman, V.P., Gillings, M., Delgado-Baquerizo, M., Zhu, Y.G. Prophage-encoded antibiotic resistance genes are enriched in human-impacted environments. Nature Commun. 2024, 15(1), 8315.

2 Supplementary Figures and Tables

2.1 Supplementary Figures

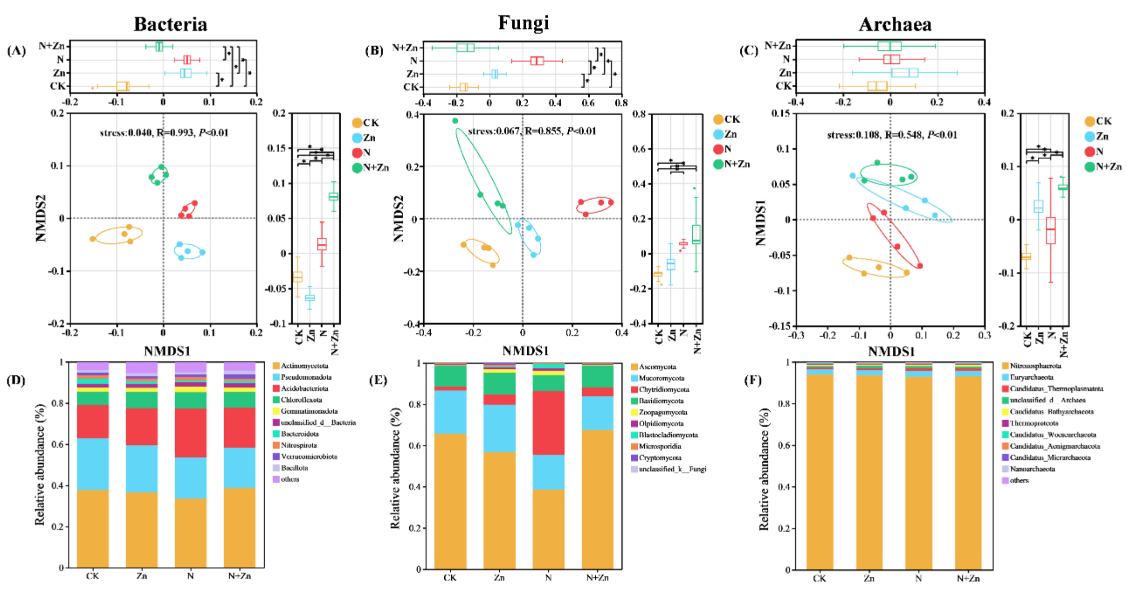

Supplementary Figure 1. NMDS analysis of the bacterial (A), fungal (B) and archaeal (C) community for visualization of Bray-Curtis distances in response to different combinations of N and Zn fertilization. Relative abundance of top 10 bacterial (D), fungal (E) and archaeal (F) phyla in response to different combinations of N and Zn fertilization.

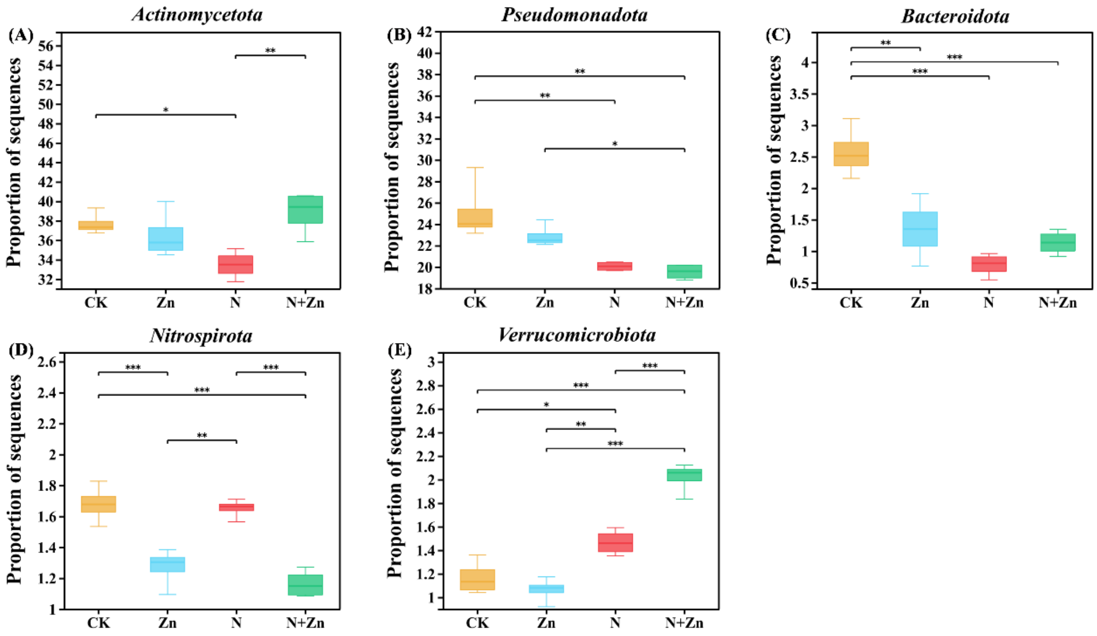

Supplementary Figure 2. Box plots of relative abundance of phyla *Actinomycetota* (A), *Pseudomonadota* (B), *Bacteroidota* (C), *Nitrospirota* (D) and *Verrucomicrobiota* (E) in response to different combinations of N and Zn fertilization based on Kruskal-Wallis H test. * indicates significance at $P < 0.05$. ** indicates significance at $P < 0.01$. *** indicates significance at $P < 0.001$.

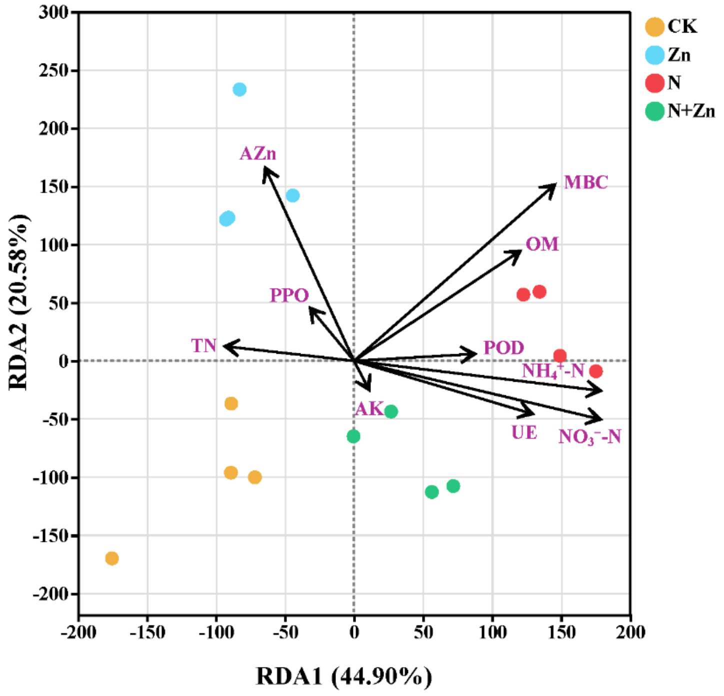

Supplementary Figure 3. RDA showing the relationships of environmental parameters with soil microbial species.

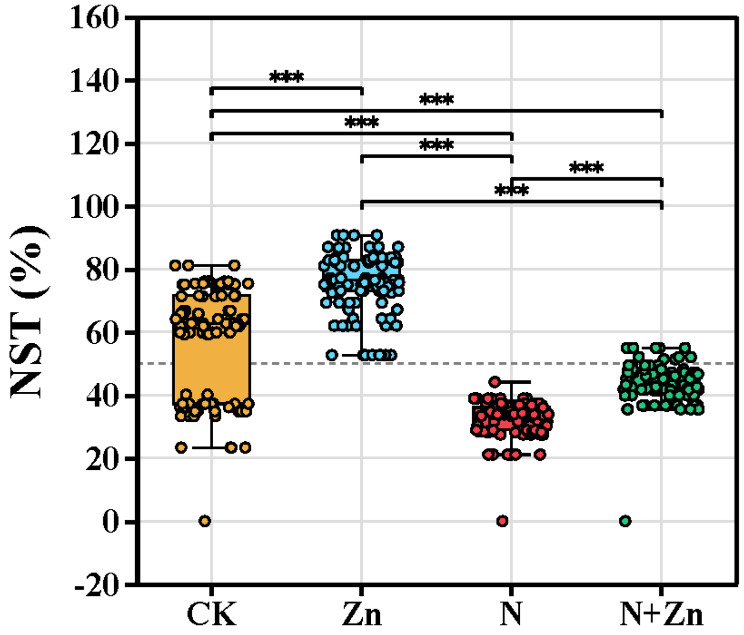

Supplementary Figure 4. Comparison of microbial community NST values based on Bray-Curtis distance in response to different combinations of N and Zn fertilization. *** indicates significance at $P < 0.001$.

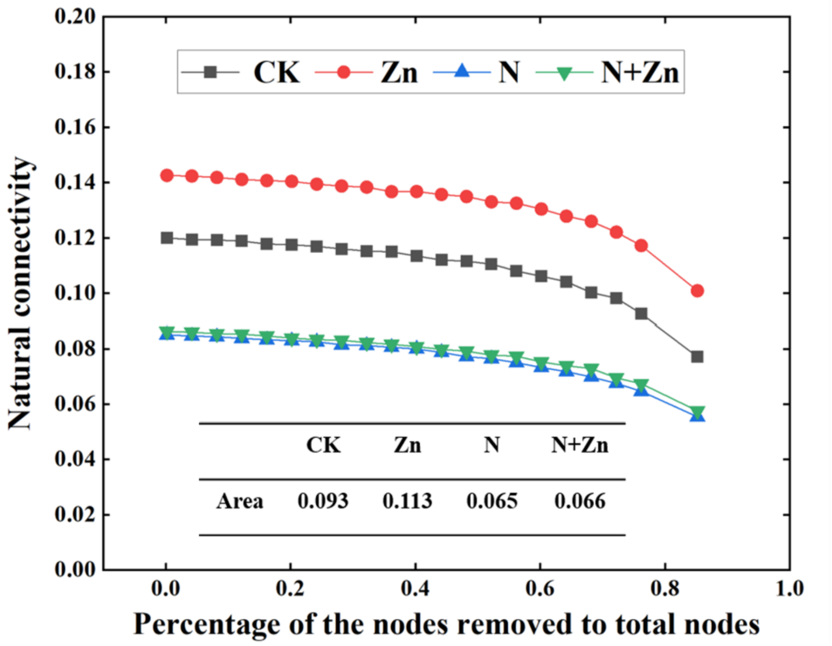

Supplementary Figure 5. Robustness curves of the co-occurrence networks under random attacks in response to different combinations of N and Zn fertilization. Natural connectivity was set as y-axis; percentage of the nodes removed to total nodes was set as x-axis.

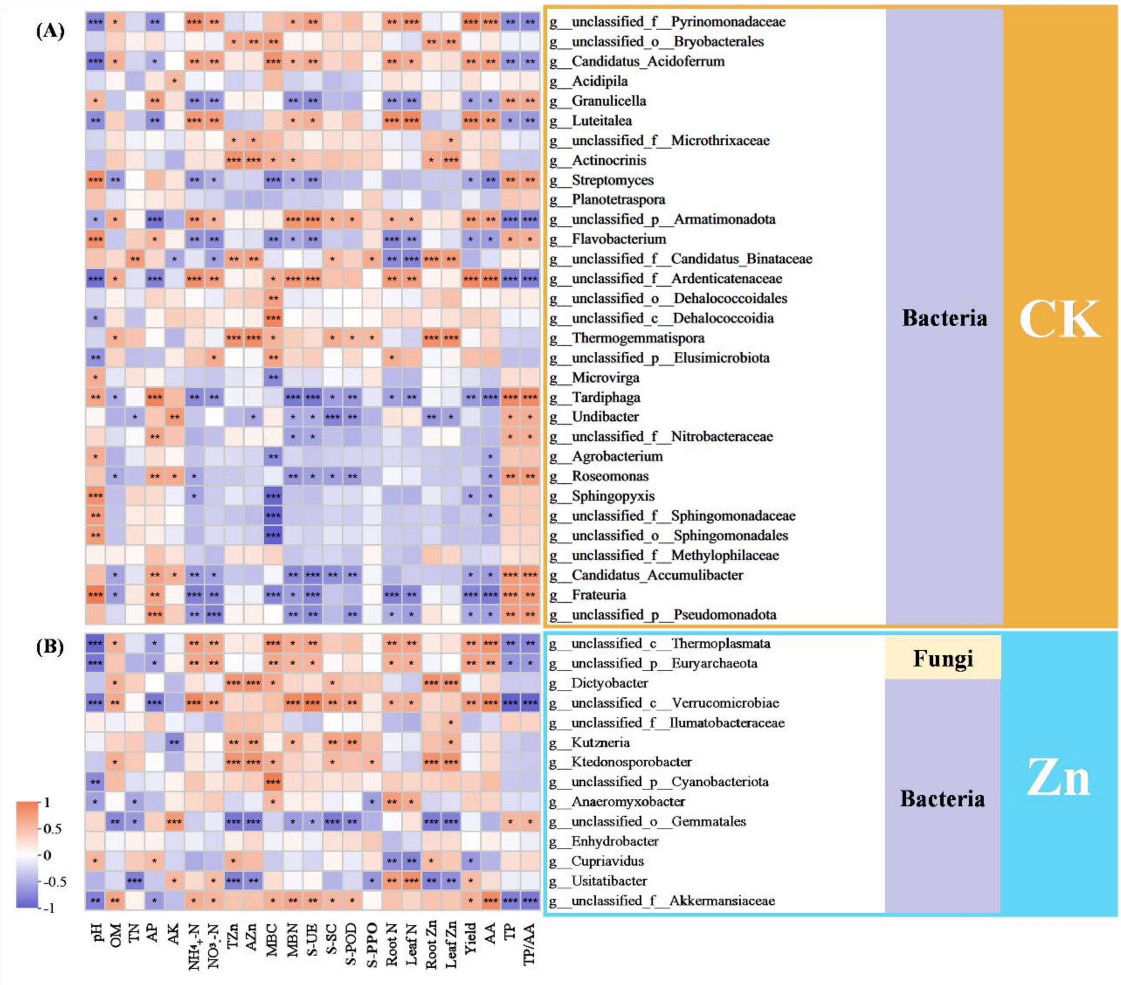

Supplementary Figure 6. The classification of key taxa in soils under CK treatment (A) and Zn treatment (B) identified by the microbial network and spearman heatmaps showing the correlations between the relative abundances of key taxa and parameters (including soil properties, soil enzyme activities and plant characteristics). * indicates significance at $P < 0.05$. ** indicates significance at $P < 0.01$. *** indicates significance at $P < 0.001$.

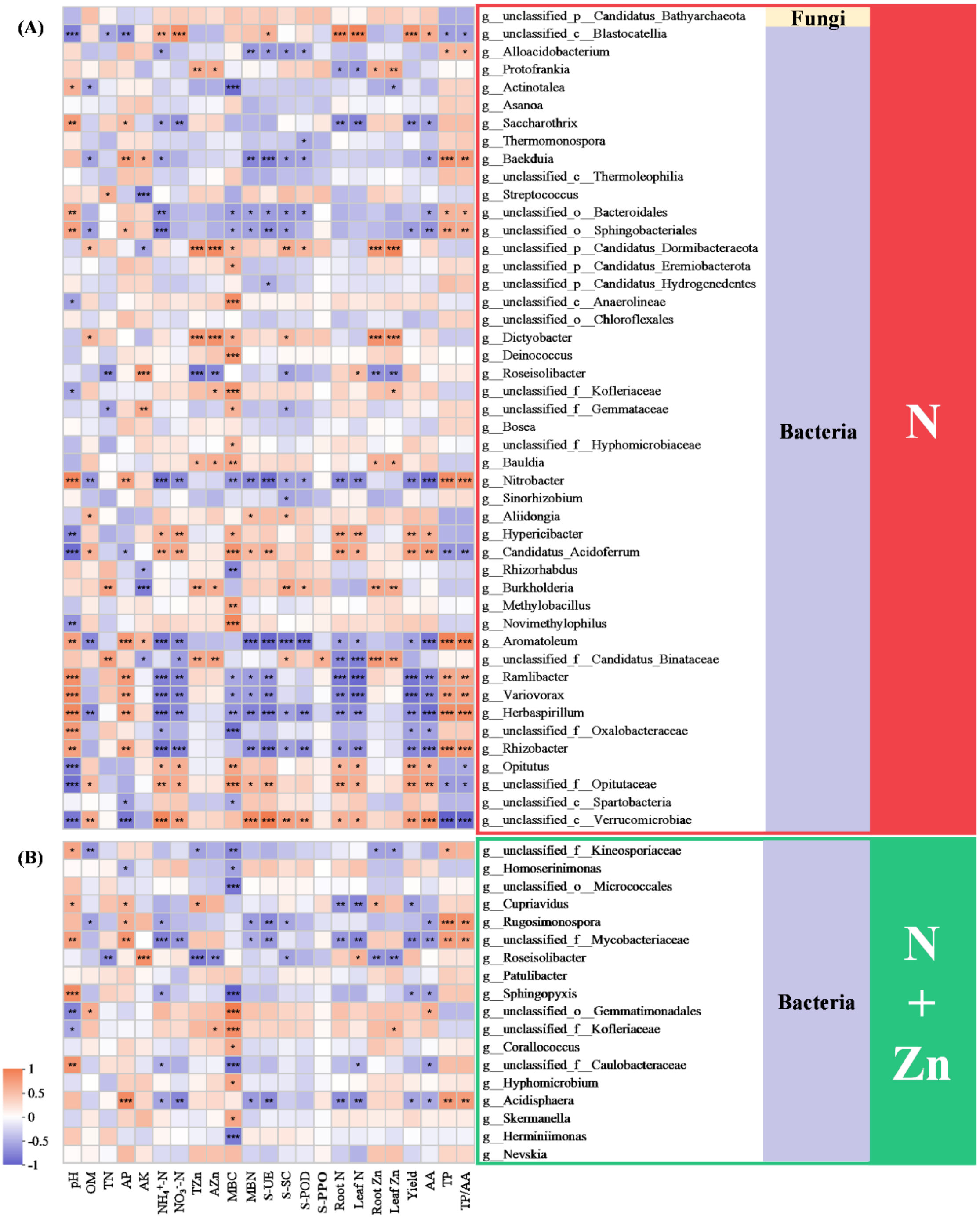

Supplementary Figure 7. The classification of key taxa in soils under N treatment (A) and N+Zn treatment (B) identified by the microbial network and spearman heatmaps showing the correlations between the relative abundances of key taxa and parameters (including soil properties, soil enzyme activities and plant characteristics). * indicates significance at $P < 0.05$. ** indicates significance at $P < 0.01$. *** indicates significance at $P < 0.001$.

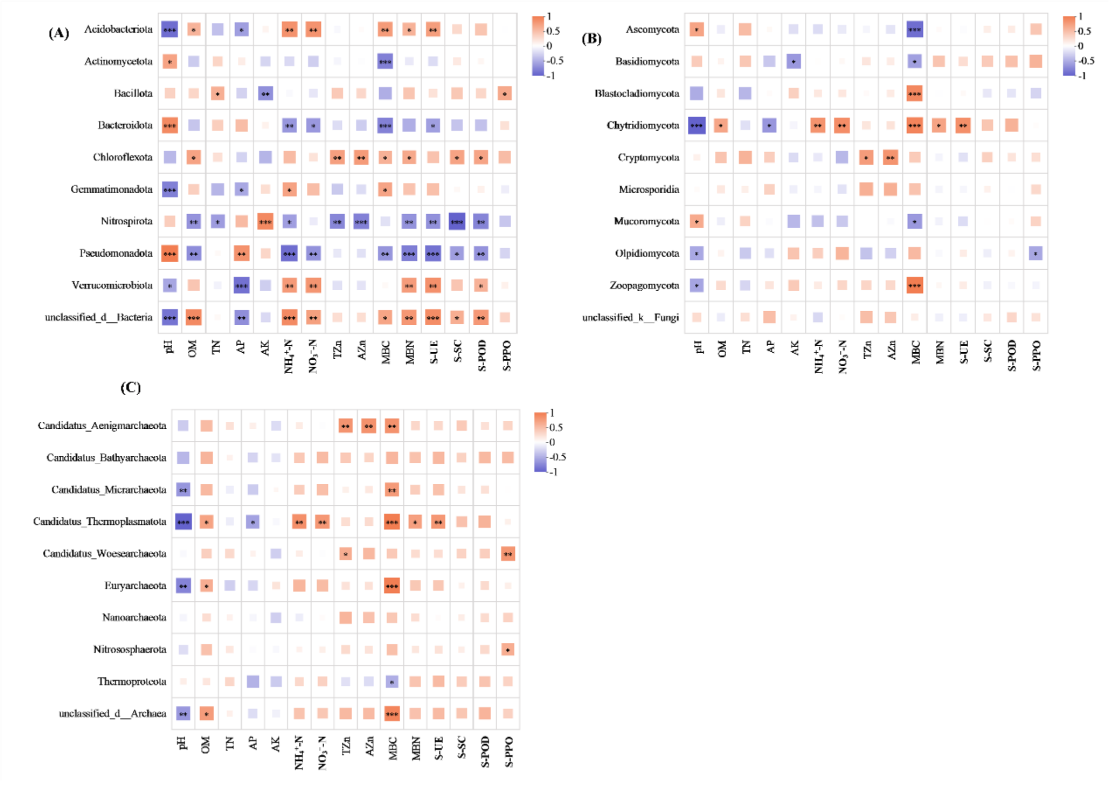

Supplementary Figure 8. Spearman correlation analysis between soil environmental variables with bacterial (A), fungal (B) and archaeal (C) taxa (top 10 phyla) in the rhizosphere soil of tea plants under different combinations of N and Zn fertilization. * indicates significance at $P < 0.05$. ** indicates significance at $P < 0.01$. *** indicates significance at $P < 0.001$.

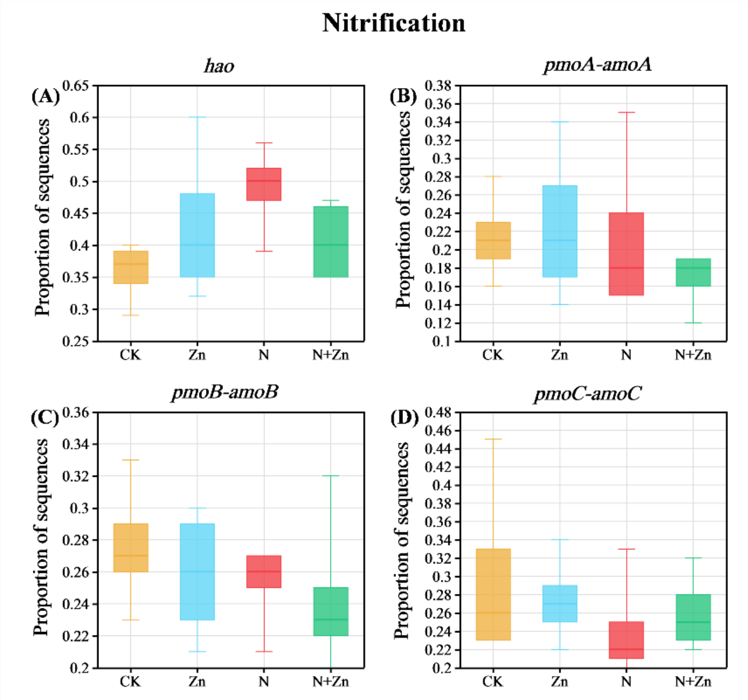

Supplementary Figure 9. Box plots of nitrification functional genes in response to different combinations of N and Zn fertilization based on Kruskal-Wallis H test. * indicates significance at $P < 0.05$. ** indicates significance at $P < 0.01$. *** indicates significance at $P < 0.001$.

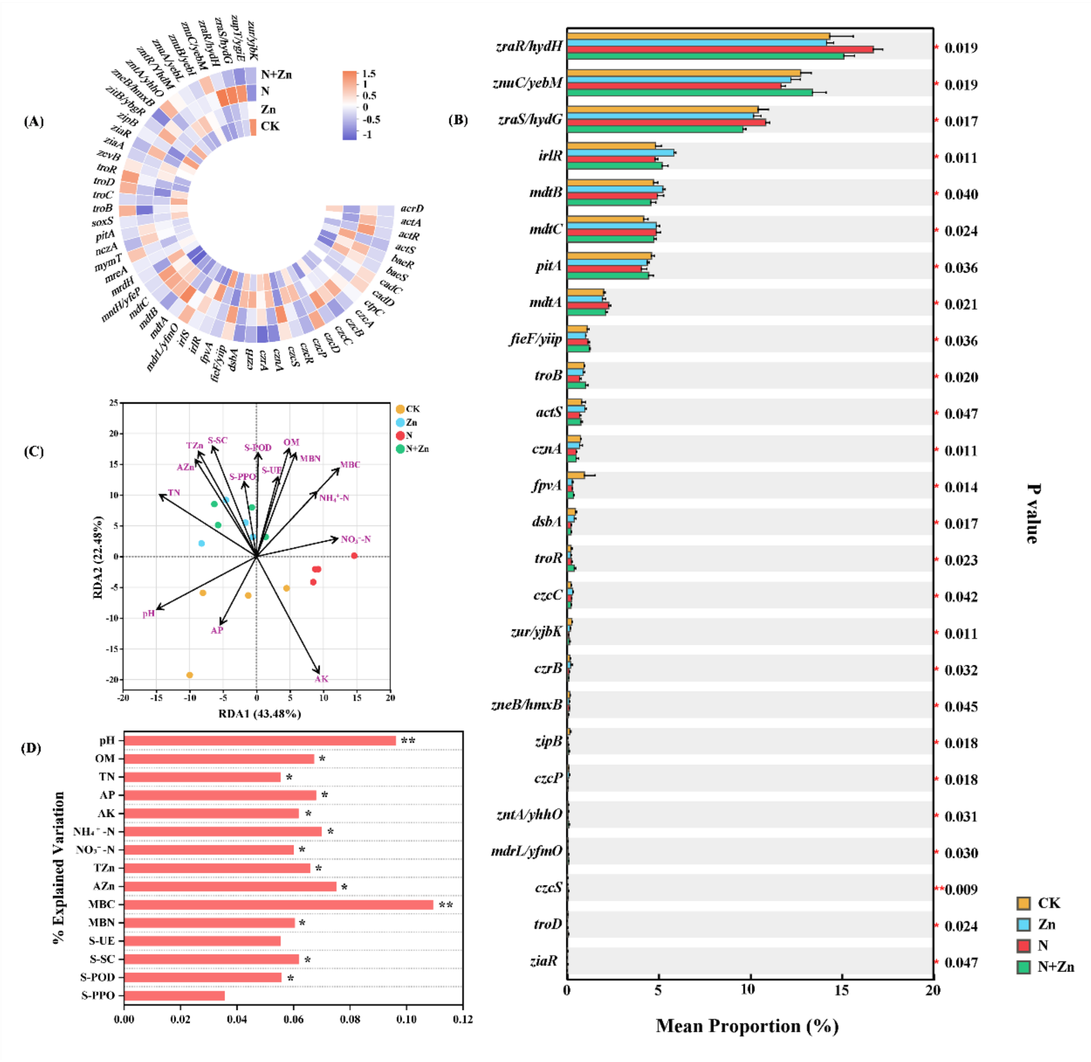

Supplementary Figure 10. Heatmaps displaying relative abundances of genes related to Zn resistance (A). Differential abundance of Zn resistance functional genes in response to different combinations of N and Zn fertilization based on Kruskal-Wallis H test (B). RDA showing the relationships of environmental parameters with Zn resistance genes (C). Hierarchical partitioning analysis and significant testing were performed to evaluate the explanatory variance and significance of various explanatory variables for genes related to Zn resistance (D). * indicates significance at $P < 0.05$. ** indicates significance at $P < 0.01$.

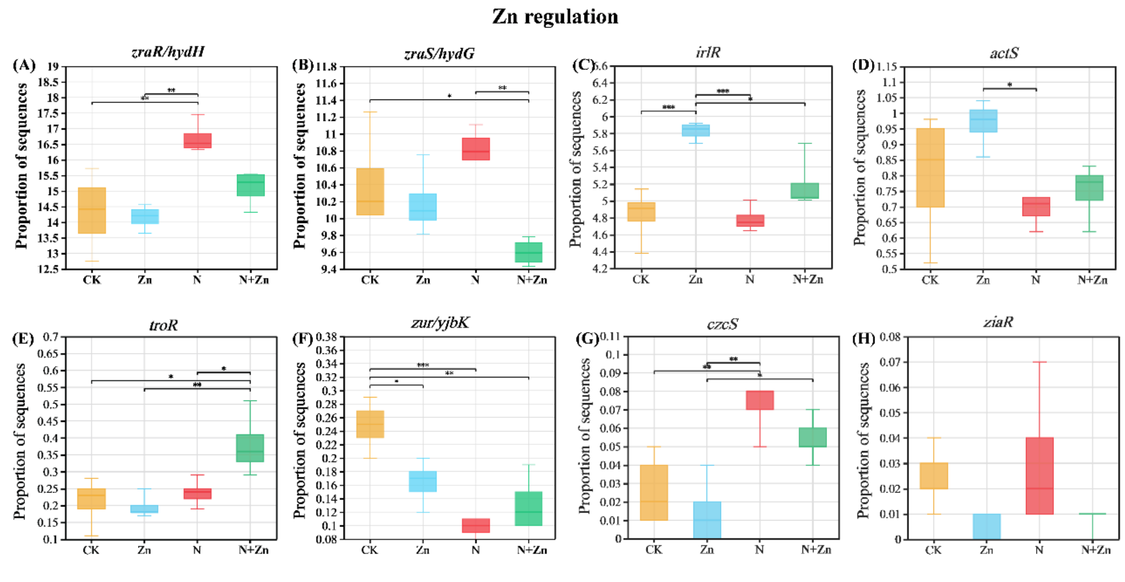

Supplementary Figure 11. Box plots of Zn regulation functional genes in response to different combinations of N and Zn fertilization based on Kruskal-Wallis H test. * indicates significance at $P < 0.05$. ** indicates significance at $P < 0.01$. *** indicates significance at $P < 0.001$.

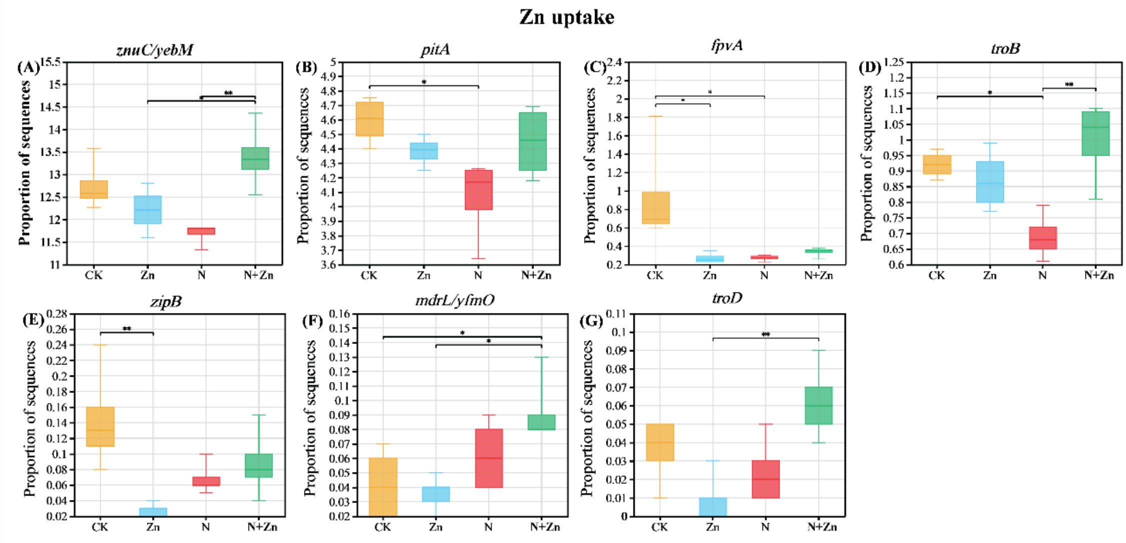

Supplementary Figure 12. Box plots of Zn uptake functional genes in response to different combinations of N and Zn fertilization based on Kruskal-Wallis H test. * indicates significance at $P < 0.05$. ** indicates significance at $P < 0.01$. *** indicates significance at $P < 0.001$.

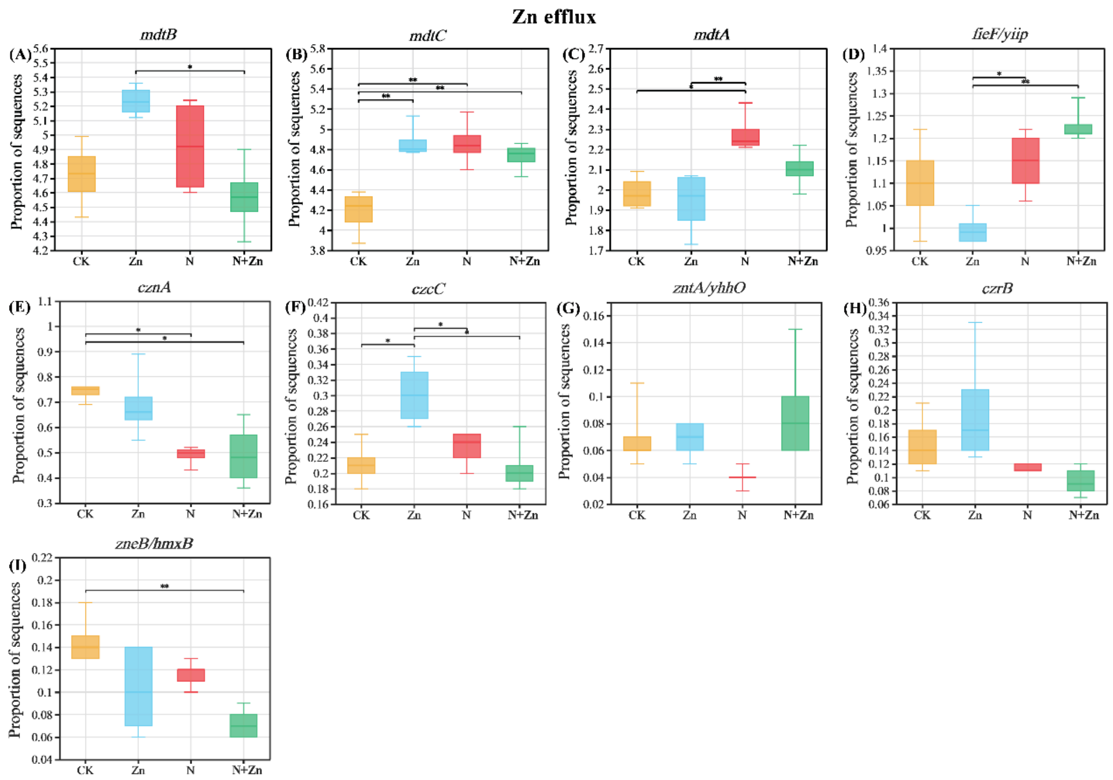

Supplementary Figure 13. Box plots of Zn efflux functional genes in response to different combinations of N and Zn fertilization based on Kruskal-Wallis H test. * indicates significance at $P < 0.05$. ** indicates significance at $P < 0.01$. *** indicates significance at $P < 0.001$.

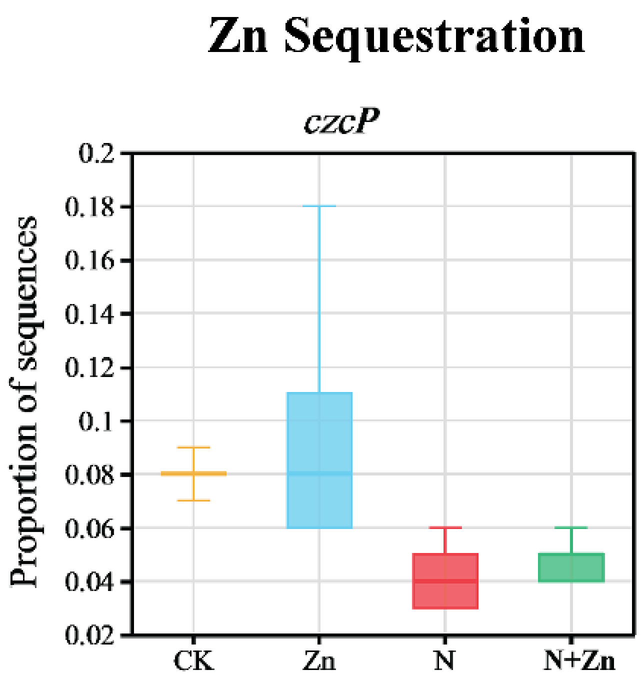

Supplementary Figure 14. Box plots of Zn sequestration functional genes in response to different combinations of N and Zn fertilization based on Kruskal-Wallis H test. * indicates significance at $P < 0.05$. ** indicates significance at $P < 0.01$. *** indicates significance at $P < 0.001$.

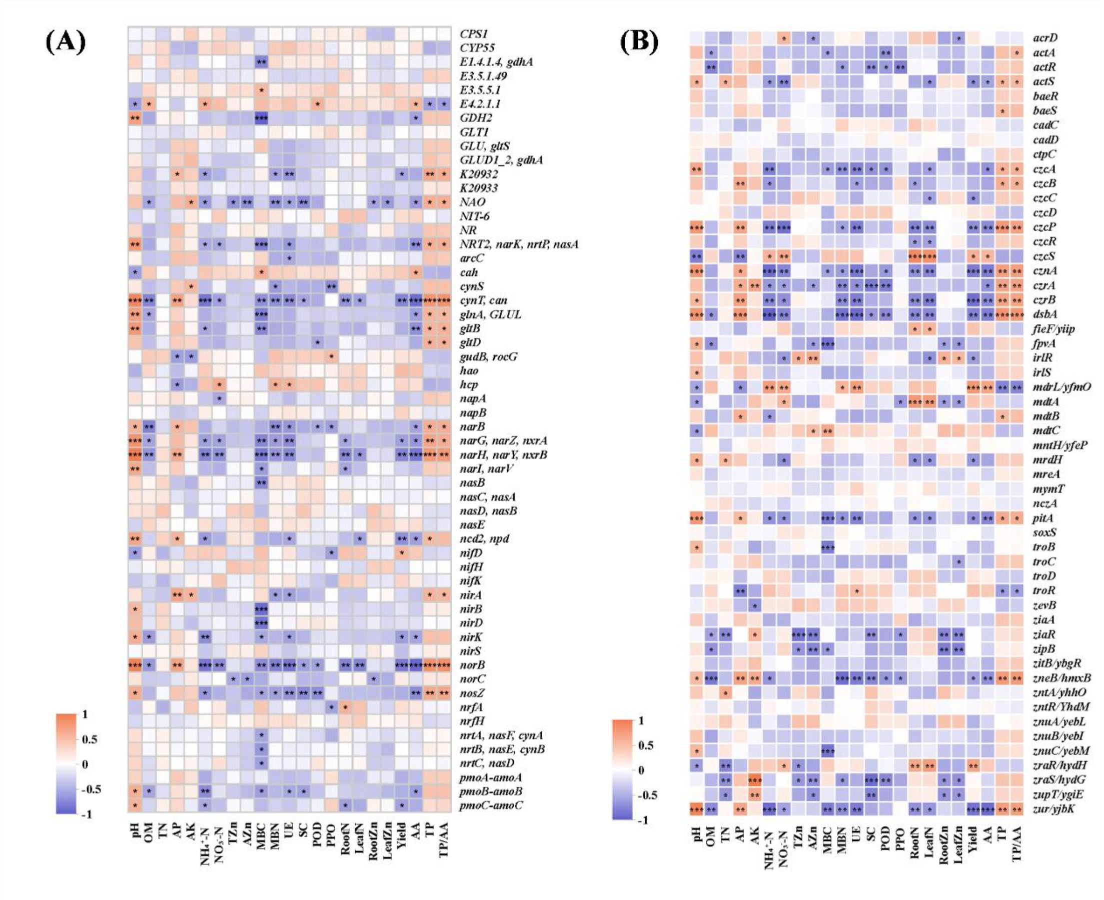

Supplementary Figure 15. Spearman correlation analysis between soil environmental variables with N cycling functional genes (A) and Zn resistance functional genes (B) in the rhizosphere soil of tea plants under different combinations of N and Zn fertilization.

2.2 Supplementary Tables

Table S1. Influences of different fertilization treatments on microbial community composition as determined using PERMANOVA based on Bray-Curtis distance metrics

	% Explained	*F*. Model	R^2	*P* value
Bacteria	80.1	16.1	0.801	0.001**
Fungi	57.7	5.45	0.577	0.001**
Archaea	54.4	4.78	0.544	0.001**

Note: *P*-value based on PERMANOVA (999 permutations). ** $P < 0.01$.

Table S2. Spearman correlation analysis between chemical properties and enzyme activities in the rhizosphere soil of tea plants under different combinations of N and Zn fertilization.

	pH	OM	TN	AP	AK	NH_4^+-N	NO_3^--N	TZn	AZn
MBC	−0.787**	0.446	−0.224	−0.190	0.096	0.500*	0.324	0.274	0.332
MBN	−0.657**	0.622*	0.132	−0.841**	−0.657**	0.759**	0.656**	0.462	0.447
UE	−0.761**	0.678**	0.116	−0.845**	−0.528*	0.855**	0.727**	0.346	0.340
SC	−0.366	0.711**	0.603*	−0.539*	−0.828**	0.594*	0.224	0.700**	0.759**
POD	−0.428	0.640**	0.426	−0.611*	−0.694**	0.621*	0.435	0.544*	0.547*
PPO	0.086	0.411	0.529*	−0.021	−0.402	0.082	−0.229	0.471	0.394

Note: * indicates significance at $P < 0.05$. ** indicates significance at $P < 0.01$.

Table S3. Spearman correlation analysis between soil environmental variables with plant characteristics under different combinations of N and Zn fertilization.

	Root N concentrations	Leaf N concentrations	Root Zn concentrations	Leaf Zn concentrations	Yield	AA	TP	TP/AA
pH	−0.760**	−0.725**	−0.078	−0.179	−0.815**	0.778**	0.772**	−0.760**
OM	0.273	0.145	0.594*	0.577*	0.465	−0.727**	−0.744**	0.273
TN	−0.435	−0.529*	0.647**	0.591*	−0.343	−0.118	−0.147	−0.435
AP	−0.667**	−0.630**	−0.009	−0.088	−0.681**	0.859**	0.853**	−0.667**
AK	0.093	0.211	−0.645**	−0.666**	0.026	0.542*	0.538*	0.093
NH_4^+-N	0.794**	0.788**	−0.229	−0.109	0.720**	−0.718**	−0.715**	0.794**
NO_3^--N	0.597*	0.624**	0.147	0.250	0.801**	−0.868**	−0.862**	0.597*
TZn	−0.265	−0.426	0.874**	0.915**	−0.150	−0.341	−0.350	−0.265
AZn	−0.232	−0.371	0.926**	0.938**	−0.081	−0.362	−0.371	−0.232
MBC	0.412	0.379	0.274	0.385	0.444	−0.391	−0.391	0.412
MBN	0.485	0.447	0.329	0.424	0.611*	−0.882**	−0.888**	0.485
UE	0.590*	0.558*	0.309	0.391	0.741**	−0.952**	−0.932**	0.590*
SC	0.115	0.044	0.706**	0.750**	0.256	−0.718**	−0.735**	0.115
POD	0.232	0.209	0.468	0.579*	0.296	−0.729**	−0.776**	0.232
PPO	−0.418	−0.344	0.462	0.532*	−0.106	−0.126	−0.162	−0.418

Note: * indicates significance at $P < 0.05$. ** indicates significance at $P < 0.01$.
